# The Added Value of Parents Practicing in Virtual Reality to Illustrate the Use of Innovative Methods in Parent-Child Interaction Therapy: Single-Case Experimental Design

**DOI:** 10.2196/60752

**Published:** 2025-07-23

**Authors:** Iza C A Scherpbier, Mariëlle E Abrahamse, Samantha Bouwmeester, Robert G Belleman, Arne Popma, Ramón J L Lindauer

**Affiliations:** 1 Child and Adolescent Psychiatry Amsterdam University Medical Center Location University of Amsterdam Amsterdam The Netherlands; 2 Academic Center for Child and Adolescent Psychiatry Levvel Amsterdam The Netherlands; 3 Mental Health Amsterdam Public Health Amsterdam The Netherlands; 4 Child Care and Protection Board Ministry of Justice and Security The Hague The Netherlands; 5 Department of Developmental Psychology University of Tilburg Tilburg The Netherlands; 6 Computational Science Lab University of Amsterdam Amsterdam The Netherlands; 7 Amsterdam University Medical Center location Vrije Universiteit Amsterdam Child and Adolescent Psychiatry & Psychosocial Care Amsterdam The Netherlands

**Keywords:** Parent-Child Interaction Therapy, virtual reality, single-case experimental design, innovation, parenting

## Abstract

**Background:**

Throughout years of research, the well-known behavioral parent training program, Parent-Child Interaction Therapy (PCIT), has been adapted and enhanced to tailor the treatment to the needs of families in community-based clinical care. This study wished to evaluate an add-on to PCIT that could be engaging for parents. As a way to enlarge practice opportunity and potentially allow parents to achieve positive treatment effects sooner, this study added virtual reality (VR) to PCIT.

**Objective:**

This study aimed to increase positive parenting skills at a faster pace with the use of PCIT-VR, on the basis that practicing positive parenting skills in the VR tool would increase parents’ overall practice time, thus leading to more confidence in their skills, which could consequently increase the pace of skill acquisition. Furthermore, we hypothesized that due to the overall increase in positive parenting skills, PCIT treatment effects such as diminishment of child disruptive behavior and parenting stress would decrease at a faster pace when VR was introduced.

**Methods:**

Families were recruited from a specialized child and adolescent psychiatry clinical practice in the Netherlands. Using a single-case experimental design, 11 families, equating to 18 participants, signed informed consent forms and received the staggered introduction of VR to treatment. As is common with a single-case experimental design, visual inspection analyses and randomization tests were conducted. Group differences were evaluated with nonparametric tests and reliable change indices.

**Results:**

Overall, our study reaffirmed that PCIT is an effective intervention for this population as there were positive treatment effects found in almost all cases. Nevertheless, there did not seem to be a clear relationship between the use of the VR tool and PCIT treatment effects, although positive parenting skills seemed to increase when VR was introduced to treatment for some parents. For all parents, questions and comments decreased with the introduction of VR. These findings tentatively suggest that practicing with VR could potentially increase positive parenting skills and also have an impact on other treatment-related outcomes, such as child disruptive behavior and parenting stress.

**Conclusions:**

This was the first time that PCIT has been supplemented with VR. We provide preliminary evidence of its added value. We cautiously suggest that VR could provide added value to PCIT and increase confidence in parenting skills for certain parents, although there are complex factors that play into treatment success that must simultaneously be considered. These factors include parents having the motivation and mental capacity to change and the complex psychological problems some families face. Although promising, we believe that due to the novelty of our VR practice tool and the variety of results from our study, more research is necessary into PCIT-VR to draw further conclusions on its effects.

## Introduction

### Background

Behavioral parent training (BPT) programs are an effective way to treat child disruptive behavior [1,2]. It is important to intervene at a young age for children exhibiting disruptive behavior to limit adverse outcomes later in life. Child disruptive behavior can lead to higher risks of aggressive behavior, criminality, internalizing problems, and other mental health–related problems later on [3]. Moreover, if persistent, long-term problems can arise in child development, family functioning, and other broader social factors, such as in parent-child relationships or educational delays [3,4]. We know that parents are the most important people in children’s early lives [5-7], which indicates the importance of including parents in treatment. Parents should function as sensitive, responsive support figures for children. Furthermore, we know that child disruptive behavior can greatly increase parenting stress and strain the parent-child relationship [8]. Hence, BPT programs aim to diminish child disruptive behavior and parenting stress by focusing on the parent-child relationship with positive parenting skills [9].

With more than 40 years of evidence, Parent-Child Interaction Therapy (PCIT) is considered an effective BPT program for young children between the ages of 2 and 7 years and their parents, and has been widely disseminated [10]. PCIT aims to diminish child disruptive behavior and parenting stress, increase parents’ positive parenting skills, and better the quality of the parent-child relationship [1,11]. Overall, PCIT is known to impactfully improve parental responsiveness and warmth [12]. PCIT includes 2 phases, the first phase is the child-directed interaction (CDI) phase and the second phase is the parent-directed interaction (PDI) phase. In the CDI phase, parents are taught to use positive parenting skills that affirm their child’s behavior, which helps to build up the quality of the parent-child relationship. This phase lays the foundation for effective behavior change. In the PDI phase, parents are taught to set safe, consistent, and effective boundaries for their child’s behavior. In this phase, the positive parenting skills that were previously taught still play an important role, allowing the parent-child relationship to continue improving over the course of the treatment [12]. To allow families to go at their own pace, PCIT has a nontime limited character, which means that treatment is completed only when certain parenting skills are met and parents feel confident in being able to manage behavior at home without the support of therapists [11].

Throughout the years, PCIT has been adapted and enhanced to tailor the treatment to the needs of families in community-based clinical care, though, for example, shortened versions of treatment or by adding incentives [13-18]. These studies demonstrated decreases in child disruptive behavior, a reduction in reports of child maltreatment posttreatment, and more positive parenting outcomes within community-based clinical samples. Notwithstanding, these studies commonly experienced high dropout rates that suggested only around 50% of families completed treatment [18]. When specifically looking at the difference between treatment completers and treatment dropouts, several factors were found that potentially predicted the dropout. These factors associated with the dropout were a lower education level, especially in combination with a female child gender, one-parent households, and internalizing problems for the mother [19,20]. Furthermore, a 3-year follow-up study showed that treatment completers reported substantially less child disruptive behavior and parenting stress than treatment dropouts [21]. It was also found that positive parenting skills increased more and negative parenting skills decreased more between pre and posttreatment when treatment was completed [19]. In addition, a crucial aspect of improving the parent-child relationship is parents using positive parenting skills [9] and skill practice was shown to be a good predictor for treatment outcomes [22]. Taken together, these studies infer that treatment should focus on increasing practice time for parents’ skill use. This could in turn lead to better treatment outcomes, such as decreasing parenting stress and thereby dropout rates. With this in mind, this study strived to adapt PCIT in an attempt to increase skill practice at home. In other words, we attempted to tailor PCIT to the needs of this community-based clinical sample treatment receivers through creating an additional practice opportunity.

Adaptations to PCIT have previously been made with the common assumption that the treatment as is, did not fully extend to the needs of a target population, such as for clinical samples [23]. In addition, as there is a high attrition rate in community-based clinical samples, it is important to enlarge engagement opportunities for this target population and engage them in treatment right from the start. We know that positive treatment effects in PCIT can already be found if at least 4 treatment sessions are attended by families [17]. Accordingly, it could be helpful to prolong the effects of these sessions through, for example, a technological tool. In more recent years, digital adaptations, such as online PCIT and Pocket PCIT (ie, a web-based PCIT resource platform) have increasingly been used to better reach parents [12,24,25]. Pocket PCIT is a passive PCIT augmentation that provides an online and on-demand resource to families to practice CDI skills [24]. The first findings of Pocket PCIT indicated that the CDI phase was completed in fewer sessions when families had access to Pocket PCIT, although they did not complete the entire treatment in fewer sessions [12]. Moreover, parents were open to completing additional homework in the form of Pocket PCIT, provided that the content was engaging, which indicates a high satisfaction rate with such a digital add-on [25]. Nonetheless, treatment-related outcomes such as child disruptive behavior and parenting stress did not differ significantly from standard PCIT, suggesting that there are only partial benefits gained from this on-demand resource [25]. This study wished to similarly create an add-on to PCIT that could be engaging for parents, but we decided to augment PCIT in a different way. As a way to enlarge treatment engagement through practice opportunity and potentially allow parents to achieve positive treatment effects sooner, this study sought to add virtual reality (VR) to PCIT [26]. VR can provide a sense of realness through immersion, which watching a video cannot. Therefore, practicing in VR would allow parents to not only be probed to think about the skills (similarly to previous digital add-ons), but it would also allow them to virtually experience using the skills [27].

In recent years, there has been an extreme increase in the use of VR in mental health care [28]. VR has been developed for new treatments but has also been used to boost or modernize treatments [29,30]. Among others, it has increasingly been implemented as a tool for internalizing problems in adults and children, such as the treatment of phobias, posttraumatic stress disorder, eating disorders, and anxiety disorders [31,32]. In these studies, VR has shown promising results with effect sizes varying from moderate to large [31,32]. Most commonly, VR has been used for exposure therapies and skills training [33]. Specifically, when VR was used as a skills trainer, a meta-analysis showed positive results in creating practice opportunities to increase knowledge-based skills [34]. Furthermore, a meta-analysis that supported the addition of VR to a variety of psychological treatments showed promising results and that it has the potential to support clinical populations [33]. In line with these findings, this study investigated the added value of VR to PCIT in a community-based clinical sample as a way to increase their practice opportunity [26].

Due to the novelty of the VR tool and the fact that PCIT is measurement-driven and a nontime limited intervention, we used a single-case experimental design (SCED) to assess the added value of VR to PCIT. Using an SCED to evaluate a novel tool has multiple advantages. First, it is perfect to use in clinical practice as it can take into account individual changes because it compares the participant with him or herself [35]. More specifically, this can help take into account the variation of problems that are seen in a highly specialized clinical mental health care sample. Second, in an SCED, there are repeated measurements of the independent variable for one individual in different treatment phases, instead of evaluating the independent variable by assigning multiple individuals to different treatment groups. Third, in addition to prepost measurement strategies, an SCED can extensively examine the impact of an intervention due to its repeated measurements approach, not only within an individual, but also through repetition across participants. This allows for an insight into how, why, and when change happens during the intervention, which is done both individually and on a group level [36].

### Objective

Therefore, through this SCED study, we evaluated the added value of VR to PCIT. Aside from measuring the effectiveness of PCIT in a community-based clinical sample, we took a closer look at individual patterns and how the staggered introduction of VR could influence treatment-related outcomes. We expected that PCIT-VR would increase positive parenting skills at a faster pace and increase treatment engagement. This is because if parents practiced positive parenting skills in the VR tool, we hypothesized that their overall practice time would increase, which could consequently increase the pace of skill acquisition. Moreover, we expected that with the addition of the VR tool, the scope would be enlarged (eg, it could be beneficial for split families with separated parents), as it provided the opportunity to practice in the absence of their child. Furthermore, we hypothesized that due to the overall increase in positive parenting skills, PCIT treatment effects such as diminishment of child disruptive behavior and parenting stress would also decrease at a faster pace when VR was introduced. Finally, we expected that the effects of PCIT-VR would be further maintained and engrained in the long run due to the additional skill training provided by our VR tool.

## Methods

The Single-Case Reporting Guideline in Behavioral Interventions (SCRIBE), 2016 was followed to explain the design, methodology, and the results of this study [37,38].

### Design

#### Type of Design

**Figure 1:**
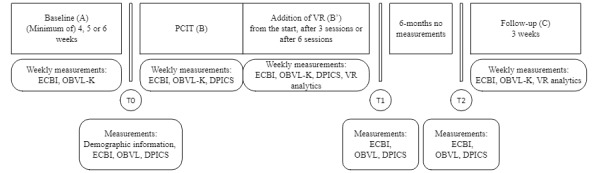
Set up of the design with phases and measurements per phase. DPICS: Dyadic Parent-Child Interaction Coding System; ECBI: Eyberg Child Behavior Inventory; OBVL-K: Dutch Parenting Stress Questionnaire-shortened version (Opvoedingsbelastingvragenlijst-Kort); PCIT: Parent-Child Interaction Therapy; T0: pretreatment measurement; T1: posttreatment measurement; T2: follow-up measurement; VR: virtual reality.

A randomized, nonconcurrent, multiple-baseline SCED across single participants (Figure 1) was applied. Intersubject replication, with weekly repetition, was used in this study [37]. Participants completed weekly measurements during a baseline phase (phase A, a minimum of 4, 5, or 6 measurements), an intervention period (phase B [PCIT] and phase B’ [PCIT with the addition of VR]), and a follow-up phase (phase C with 3 measurements) that took place 6 months posttreatment. Between phases, a more extensive measurement took place as a way to assess pretreatment, posttreatment, and follow-up group differences as well (Figure 1). Researchers, therapists, and participants were not blinded to any components of the study. Nobody was blinded because researchers had to administer the VR component at the correct time, therapists could refer to the VR component during treatment, and participants were allowed to know when they would receive the add-on.

#### Randomization

Participants were randomized twice at the start of the study (Figure 1). As the waitlist for the treatment typically varied between 4 to 12 weeks, participants were randomized to phase A (ie, baseline) for a minimum of 4, 5 or 6 weeks, which means a multiple baseline design was used. Participants were contacted for participation while on the waitlist to start treatment. In this study, participants never remained on the waitlist longer to finish phase A, meaning clinical practice was not obstructed. Moreover, to allow for random sequencing of the intervention phase, the reception of VR was also randomized. Participants received VR either straight away, after 3 sessions, or after 6 sessions. This way, we could assess the added value of VR as we expected to see a direct difference in treatment results when VR was added. This limited the possibility of change occurring due to maturation and we could most relevantly attribute change to VR. In other words, both the baseline and intervention randomizations were done to control for potential time-related confounders [39]. The randomization of phases A and B/B’ was done by a noninvolved researcher from the department, who would let the researcher know what randomization the participants had received. Phase C was not randomized, as all participants were contacted 6 months after treatment.

#### Procedural Changes

The original study protocol stated that 15 children would be included [26]. However, due to factors such as the COVID-19 pandemic; attrition; and losing therapists to, for example, other jobs; we were unable to include 15 children who completed all measurements. However, with an SCED, the amount of data per phase (ie, at least 3 to 5 data points) per participant are more important than the number of participants, as participants are merely compared within themselves. This amount of data per phase were adhered to in most cases. Refer to the *Participants* section for the final inclusion number and number of data points per phase per participant.

Moreover, in the original study protocol, there were no weekly measurements incorporated in the follow-up phase; this phase originally consisted of 1 single follow-up measurement. However, the third author (SB), who has expertise in SCED analyses, suggested adding additional weekly measurements to be able to compare that phase with previous phases. Accordingly, an amendment was written and subsequently granted by the medical ethical committee. This meant participants who had previously joined the study were asked to sign another informed consent form if they agreed to have 3 additional weekly measurements in phase C after the single follow-up measurement (Figure 1).

### Participants

#### Participants Characteristics and Selection Criteria

Participants were recruited from a specialized child and adolescent psychiatry clinical practice in the Netherlands, after being referred through community channels. For the full selection procedure, please consult the study protocol [26]. The inclusion criteria for the study were as follows: (1) child disruptive behavior problems were a reason for referral, (2) children were aged between 2 and 7 years old, and (3) parents spoke Dutch or English. Participants were excluded from participation if any of the following criteria were met: (1) a child had a severe physical impairment, such as deafness; (2) a child had a mental disability (IQ <60); (3) an unsafe home situation, where home displacement was indicated; (4) a child or parent with problems requiring personal health care, such as suicidality, or parents with aggression regulation or addiction problems; and (5) parents known to have severe problems with motion sickness.

In total, 23 participants were screened to participate, of which 20 (87%) were approached as they met inclusion criteria. A total of 18 (78%) parents of 11 children were willing to participate in the study. Participants signed informed consent after receiving verbal and written information about the study, and after being able to ask all questions regarding the study to the researchers. If there was no wish to participate in the study, families would receive treatment as usual (PCIT without the addition of VR). A total of 1 (11%) couple dropped out during baseline. In total, we obtained data from all phases from 6 (33%) participants. The remaining 10 (56%) participants missed data in at least 1 phase of a measurement point, but were included in the analyses, nonetheless. In the *Results* section, we will go into detail on reasons for dropout per family.

### Context

#### Setting

The specialized child and adolescent psychiatry clinical practice in the Netherlands used in this study has multiple locations in and around Amsterdam. Depending on where participants lived and what team they were referred to, they were in clinical care in 1 of 3 locations. Another option was that they received treatment in-home, which was the case for 3 (%) families. All sessions were conducted by certified PCIT therapists according to PCIT International criteria. All pretreatment, posttreatment, and follow-up observations were conducted in-home by the first author (ICAS), and all the questionnaires were obtained digitally.

### Ethical Considerations

This study was approved by the independent medical ethics committee of the Academic Medical Center of Amsterdam, the Netherlands (2020_143/NL74210.018.20). Furthermore, the principles of the World Medical Association Declaration of Helsinki [40], and the Medical Research Involving Human Subjects Act were adhered to.

Written informed consent was obtained from participants before the study and participation was voluntary. Data were anonymized. Participants received no compensation for participation apart from being able to keep the VR headset placeholder.

### Intervention

#### PCIT Treatment

PCIT is an evidence-based treatment for young children with disruptive behavior problems aged between 2 and 7 years and their parents [1,11]. During the treatment, which consists of 2 phases, parents are coached live by therapists from behind a one-way screen through an earpiece. In the first phase, the CDI phase, parents follow their child’s play while being coached to use child-centered interaction skills. In this phase, parents are being taught to use positive parenting skills, which include labeled and unlabeled praises, reflections, and behavior descriptions, as this positively reinforces the behavior their child is displaying. Parents are also taught to limit using questions, commands, and criticisms, as this can negatively impact the parent-child interaction. Ultimately, using positive parenting skills and limiting unwanted verbalizations leads to an improvement in the parent-child relationship. The second phase, the PDI phase, is reached when parents achieve the set amount of child-centered verbal skills (statements including 10 labeled praises, 10 reflective statements, and 10 behavior descriptions, and no questions, commands, or criticisms). During the PDI phase, parents learn to set effective and safe limits for their children. They are taught to give effective commands and how to respond consistently to their child’s compliance and noncompliance. This allows for their child’s behavior to be structured in a developmentally appropriate way. The treatment requires for parents to practice “special time” with their children 5 minutes every day at home. PCIT does not have a set number of sessions, parents rather progress at their own pace and naturally reach a subsequent phase when meeting the required skill criteria.

#### VR Component

As an additional skill training tool, parents could practice with VR at home. The VR component consisted of prerecorded 360-degree videos that portrayed a child actor playing with PCIT-appropriate toys in a PCIT playroom. The video showed the child playing, after which the fragment stopped and called for the parents to respond to a multiple-choice question. An instruction video explaining how the VR environment worked was optionally available to watch before starting to practice. In a VR practice session, parents were posed statements so they could practice positive parenting skills taught in PCIT (ie, labeled praises, reflective statements, behavior descriptions, and ignoring unwanted behavior). An example was “Describe her behavior,” for which they had 2 options to answer with, such as “You put the yellow block on the green block*,”* or “Put the block on the tower*.”* An answer would only be selected when the pointer circle visible in the VR environment would increase in size for 3 seconds, as a way to warn participants about their imminent selection. Moreover, the selection was based on head movement rather than eye movement. After having selected an answer, parents received feedback on their respective answers. In the aforementioned example, if they selected the first option, they would see the following feedback: “That was a good behavior description.” If they selected the second option, they would see, “That was a command. Try to describe her behavior. For example, ‘you put the yellow block on the green block.’” The video resumed to the next fragment showing the child playing. The more the parents selected the correct answers that depict a positive parenting skill, the less examples they were offered of that particular positive parenting skill. Parents received more elaborate feedback (such as in the aforementioned example) if they did not select correct answers. After parents saw a few different scenarios, they were encouraged to practice different positive parenting skills out loud, without receiving feedback on every possibility. A VR practice session lasted approximately 10 to 15 minutes, depending on the answers given by parents.

### Measures and Materials

#### Measures

All primary measures were completed weekly. The Dyadic Parent-Child Interaction Coding System (DPICS) [41], the Eyberg Child Behavior Inventory (ECBI) [42,43], and the Dutch Parenting Stress Questionnaire [44] were also completed before treatment, after treatment, and at 6 months follow-up. All measures apart from the DPICS were digitally obtained via Castor Electronic Data Capture (Castor Research Inc), which is a secure data management platform [45]. Demographic information was filled out solely at pretreatment.

#### Demographic Information

Parents were required to fill out a questionnaire regarding background information about their child and his or her family. Information regarding sex, age, origin, family composition, parental work situation, and education level were included.

#### DPICS Tool

The DPICS [41] is a reliable and valid behavioral observation coding system. It evaluates the quality of the parent-child interactions by coding open verbal and physical behaviors of both parents and children. The following DPICS categories were used: labeled praise, unlabeled praise, reflection, behavior description, question, direct command, indirect command, and negative talk. These were subsequently categorized into the following 2 categories for the CDI phase: “positive following,” which consists of labeled praise, unlabeled praise, reflection, and behavior description, and “negative leading,” which consists of question, direct command, indirect command, and negative talk. To code with the DPICS, researchers must be trained to be able to achieve an 80% agreement rate on scored behaviors. All observations were transcribed verbatim to monitor interrater reliability. In this study, there are 2 ways the DPICS was used. First, researchers used the DPICS during the pretreatment, posttreatment, and follow-up assessment. Here, coding was done through 3 standard 5-minute play situations (child-led play, parent-led play, and clean-up). There was an average of 86% agreement between coders. Second, therapists scored a weekly 5-minute standardized observation moment at the start of every CDI session to monitor skill acquisition.

#### VR User Analytics

Each parent received a personalized link that automatically registered their use and progress in VR. The number of times (per week) that VR was used was monitored by the first author (ICAS) through a secure platform.

#### ECBI Questionnaire

The ECBI [42] is a questionnaire that addresses child disruptive behavior for children aged 2 to 16 years and is filled out by parents. The ECBI consists of 2 scales—one measuring the frequency of the problem behavior (Intensity scale: 7-point scale from 1 [never] to 7 [always]) and another that records the extent to which parents experience the behavior as a problem (problem scale: yes or no). The psychometric properties of the Dutch-translated version of the ECBI are good [43,46]. The ECBI was filled out weekly, in addition to being filled out at pretreatment, posttreatment, and follow-up assessments.

#### Dutch Parenting Stress Questionnaire (Shortened Version)

The Dutch Parenting Stress Questionnaire (Opvoedingsbelasting vragenlijst-Kort [OBVL-K]) [44] contains 34 questions that address 5 aspects of parenting stress. These are problems in the parent-child relationship, problems in parenting, depressed moods, role restriction, and health complaints. The total parenting burden score is calculated with a T-score. The psychometric properties of the questionnaire are good [44]. In the shortened version of the OBVL (ie, OBVL-K), there are 10 questions about the different aspects of parenting that together give a total score on parenting burden. The OBVL-K is used to assess weekly parenting burden, whereas the normal version is used before treatment, after treatment, and at follow-up.

#### VR Equipment

A head-mounted display that can house a smartphone was bought for all participants [47]. This study chose to use 360-degree videos that could be played on a smartphone device. This meant that participants could place their device into the headset and look around 360 degree from a singular point in the virtual environment, which in technical terms refers to 3 degrees of freedom. In other words, users could move their heads in all directions, simulating a natural look around, but did not have the capability of moving in a direction within the virtual space. This form of VR was inexpensive, easy to use with a protected personalized weblink, and allowed participants to practice in the comfort of their own homes. The videos were streamed over a network and played on a web browser, which allowed almost all devices with access to internet to be able to run the content. The raw videos were edited into fragments with key events that showed a multiple-choice question at the end of each fragment. The answer was selected by looking at the answer box with a built-in eye bullet for 3 seconds. The fragments were given in a nonlinear sequence, determined by the user’s responses. This was done in a web-based scenario editor created by ICAS, the fourth author (RGB), and bachelor and master students from technology-based studies [48-52]. Before inclusion, a small amount of preliminary work was done. The product was tested by therapists before full development and use. Nonetheless, this study is the first to explore the addition of VR to PCIT with clinical participants.

#### Procedural Fidelity

Adherence to the treatment procedure was accomplished using the standard PCIT protocol for treatment sessions. Furthermore, ICAS was responsible for sending weekly questionnaires, which could be automatically done electronically via Castor Electronic Data Capture. A data monitor appointed by the Amsterdam University Medical Center was appointed to monitor the process with 2 monitoring visits throughout the project. The observational parts of the pretreatment, posttreatment, and follow-up assessments were also planned and coordinated by ICAS with the families. Although they received weekly reminders to fill out the questionnaires and practice with VR, it was the parents’ responsibility to do this at home.

### Analyses

#### Overview

Visual inspection analyses and randomization tests were conducted on a shiny app Single Case Design website [53]. Other analyses were conducted via R Statistical Software (version 4.2.1; R Core Team 2022) and SPSS Statistics (version 28; IBM Corp) [54]. We split the analyses up into 2 parts. First, we assessed the treatment effects of PCIT. Second, we analyzed the effects VR had on treatment outcomes.

#### Primary Measures

To evaluate the effects of the treatment per person, we conducted visual inspection analyses per person for the ECBI, OBVL-K, and DPICS. We expected to find that scores would decrease on the total score of the ECBI Intensity Scale and the T-score of the OBVL-K over time. For the DPICS, we expected an increase in positive following and a decrease in negative leading over time. Second, to evaluate the effect of the intervention on ECBI Intensity Scale and OBVL-K scores, randomization tests were performed complementarily to statistically test the difference in means [55,56]. First, for each individual participant the effect size, Cohen *d,* was calculated. Next, 1000 random samples were drawn from the individual scores, and for each of these samples, the Cohen *d* was calculated. The *P* value for the individual was defined as the number of random samples that had an effect size Cohen *d* that was as large or larger than the observed Cohen *d* divided by 1000. To calculate *P* value for the group, the sum of the individual *P* values was calculated. The *P* value for the group was defined as the probability of finding the sum of *P* values given that the H0 (null hypothesis) distribution where the individual *P* values were uniformly distributed between 0 and 1. We calculated the overall *P* value for both the entire group and for the group of treatment completers.

Furthermore, to assess the added value of VR, vertical lines indicating the start of when parents received the addition of VR were added in the visual inspection analyses of the DPICS. We expected to find that when VR was added to treatment, the next measured positive following score would be higher and the next measured negative leading score would be lower. Therefore, we evaluated the hypotheses that the mean and median difference score between positive following before and after the use of VR was positive, while the mean difference score between negative leading before and after the use of VR was negative.

#### Secondary Measures

We secondarily evaluated the mean group differences from pretreatment to posttreatment and to follow-up in the DPICS categories of positive following and negative leading through a nonparametric Wilcoxon signed rank test. Effect sizes were calculated with Cohen *d*. We expected to find that positive following would significantly increase from pretreatment to posttreatment and remain stable at follow-up. Similarly, we expected to find that negative leading would significantly decrease from pretreatment to posttreatment and remain stable at follow-up. To evaluate DPICS per person, we evaluated their raw scores from pretreatment to posttreatment and to follow-up. Furthermore, reliable change indices (RCIs) were calculated to illustrate the change in the total scores of the Intensity and Problem scales in the ECBI and the total T-score on the OBVL-K from pretreatment to posttreatment and to follow-up. We expected to find that all 3 would reliably diminish from pretreatment to posttreatment and remain stable at follow-up. We expected scores to be less than the clinical range at posttreatment and at follow-up.

## Results

### Overview

First, we described the sample population with demographics, description per family, treatment trajectory, and research participation. Second, we analyzed the primary measures through visual inspection analyses and randomization tests. Third, we reported the secondary measures from pretreatment to posttreatment and to follow-up. We examined group outcomes of PCIT to show the effectiveness of the treatment for this entire sample. We looked at how often parents practiced with VR and what their DPICS scores were. In addition, we assessed their ECBI and OBVL scores through RCI.

### Demographic Sample Population

#### Overview

To gain an understanding of the sample characteristics, see Table 1 for the relevant demographic information per participant. Moreover, Multimedia Appendix 1 presents an overview of per participant at baseline and VR randomization, what measurements were completed, and how many measurements were missed per phase as per the SCRIBE guidelines [37,38]. No participants reported adverse events during the study.

**Table 1:** Participants’ demographics showing gender, relationship, custody status, family income, and child’s gender and age.

Participant	Parent gender (per participant)	Relationship status	Custody status	Family income (€ per month^a^)	Child’s gender	Child’s age (y)
01^b^	Female	Single parent	Sole	1000-2000	Male	4
02 and 03	Male and female	Divorced	Shared	Participant 02: >5000; participant 03: 2000-3000	Female	5
04	Female	Divorced	Sole	1000-2000	Male	6
05 and 06	Male and female	Married	Shared	3000-4000	Male	3
09	Female	Single parent	Shared	4000-5000	Male	6
10^b^ and 11^b^	Male and female	Cohabiting	Shared	<1000	Male	5
12^c^	Female	Single parent	Sole	<1000	Male	5
13^b^ and 14^b^	Male and female	Married	Shared	4000-5000	Female	4
15 and 16	Male and female	Married	Shared	>5000	Female	2
17^d^ and 18^d^	Male and female	Divorced	Shared	Unknown	Male	7

^a^€1=US $1.08 on average.

^b^Dropped out fully.

^c^Dropped out of intervention but remained in the study.

^d^The information collected from casefile due to missing pretreatment assessment.

#### Description of Each Participating Family

Reasons for referral and experiences with PCIT and VR are described per participant. All cases were referred for highly specialized clinical mental health care. Details of any family dropouts or significant events are also provided. Multimedia Appendix 1 supports the information for each trajectory.

Participant 01: a single mother who participated alone in the treatment with her 4-year-old boy. The child had contact with his father once a week and had 2 half-siblings that lived under the same roof. The mother had a history of psychiatric problems and was declared unfit to work. The child was clinically diagnosed with attention-deficit/hyperactivity disorder (ADHD), overall developmental delay, and parent-child relationship problems. Reason for referral was the child’s disruptive behavior. He was impulsive, did not listen, and did not respond to punishment. The mother practiced with VR once, but indicated she was too tired to do it more often. The mother and child dropped out of treatment after 3 CDI sessions because it caused too much psychological burden and stress for the mother. She indicated willingness to be contacted for research purposes 6 months after treatment, but due to the child temporarily being placed out of home, no measurement took place. Due to dropping out, only descriptive and visual inspection was included from this family. They were excluded from pretreatment, posttreatment, and follow-up measurement analyses.

Participants 02 and 03: a divorced (with shared custody) father and mother participated together in treatment with their 5-year-old girl. The child, together with her sibling, alternated every 3 nights between her father and mother. The mother had a history of personal psychiatric problems for which she had been and still was in treatment. The child was clinically diagnosed with early relation-specific traumatic experiences, parent-child relationship problems (specifically with the mother), and disruptive behavior at home. These were also the reasons for referral. The father was positive about VR, although he experienced technological problems at first. The mother did not feel immersed in VR and stopped practicing with it due to that. Due to psychological stress, the mother only filled out questionnaires at the 6-month follow-up. Unfortunately, clinical DPICS scores were not registered for research purposes by the therapist and were therefore unavailable for this research project.

Participant 04: a single mother who participated alone in the treatment with her 6-year-old boy. Neither the mother nor the child had contact with the father. The child had no siblings. The mother had a history of psychiatric problems and had previously been in treatment. The child was clinically diagnosed with separation anxiety disorder (later in remission) and there were parent-child relationship problems. Reasons for referral were the diagnosed attachment problems and manipulative behavior of the child. They completed PCIT. The mother was positive about the results of PCIT and expressed VR helped her to constructively practice parenting skills. The therapist recorded 10 DPICS scores during the CDI phase and 2 DPICS scores during the PDI phase.

Participants 05 and 06: a married father and mother who participated together in treatment with their 3-year-old boy. The child had 1 sibling. The mother was recovering from burnout (ie, extreme physical and emotional exhaustion with mental health repercussions) at the start of the treatment. The father had a history of psychiatric problems for which he received treatment in the past. The child was clinically diagnosed with disruptive behavior and language delay. There were suspicions of autism spectrum disorder (ASD) and ADHD, but these were not officially diagnosed. The reason for referral was to lessen disruptive behavior and regain tranquility in the home environment. Upon completion of PCIT, the parents were positive about the results; their child’s language had improved because of reflective statements and they saw that positive reinforcement helped in contact with their child. Both the parents practiced with VR, especially at the beginning of treatment. Later, they felt they gained more from practicing live. Due to the parents feeling overwhelmed with psychological care for their child, they only completed questionnaires at the 6-month follow-up. Unfortunately, clinical DPICS scores were not registered for research purposes by the therapist and were therefore unavailable for this research project.

Participants 07 and 08: while on the waitlist (baseline measurements, ie, phase A), the father and mother received intermediate psychological care for their child. This first caused PCIT to be postponed, and subsequently, it was concluded that PCIT was no longer necessary. No further information is known as they did not complete the T0 measurement. They will not be further discussed.

Participant 09: a single mother (with shared custody) participated alone in treatment with her 6-year-old boy. The father was aware and consented to the treatment, but did not want to fully commit. He participated in a few treatment sessions to gain an understanding of PCIT. The child was diagnosed early on with a genetic defect that could lead to neurological consequences. He was later clinically diagnosed with unspecified ADHD and unspecified intellectual disability. Originally, the child was referred by a pediatrician to diagnose ADHD and to treat it accordingly. Besides PCIT, the child also received medication (methylphenidate and melatonin) for his concentration problems. Upon completion of PCIT, the mother was positive about the reduced disruptive behavior and she was able to set effective limits for her child. She expressed that VR helped her to learn and practice the skills during the CDI phase, but that she missed the continuity of VR during the PDI phase. Unfortunately, clinical DPICS scores were not registered for research purposes by the therapist and were therefore unavailable for this research project.

Participants 10 and 11: a married father and mother participated in treatment together with their 5-year-old boy. The child had 2 half-siblings who he did not live with. The mother was simultaneously in treatment for personal psychological problems. The child was clinically diagnosed with ADHD combined type, a language delay, and there were parent-child relationship problems. The national institution that offers advice and support on matters concerning domestic violence and child abuse (in Dutch: “Veilig Thuis”) was involved with this family due to reports of potential in-home violence. The reason for referral was to improve the parent-child relationship and to decrease behavior and parenting problems. The parents were meant to receive VR but expressed that their burden and stress were too high to fit in time to practice with VR. They successfully completed the CDI phase of PCIT, but the intervention was temporarily paused. They did not restart PCIT due to experiencing a high psychological burden. No contact was achieved for T1 or T2. The therapist recorded 7 DPICS scores from the CDI phase. Due to dropping out, only descriptive and visual inspection was included from this family. They were excluded from pretreatment, posttreatment, and follow-up measurement analyses.

Participant 12: a single mother participated alone in treatment with her 5-year-old boy. The child was an only child and there was no contact with his father. The mother has a history of psychiatric problems for which she had been in treatment. During treatment, she was recovering from a recurring hernia. The child was clinically diagnosed with a disorganized attachment style, disruptive behavior, sleeping problems, and pica. The reason for the referral was to diagnose problem behavior and subsequently treat the respective diagnosed behavior with PCIT. The child had weekend foster care every 3 weeks. The mother and child dropped out of PCIT after a few CDI sessions, as the mother felt her child’s behavior and their relationship had largely improved. Overall, the mother was positive about the treatment. She attempted practicing with VR but experienced nausea and dizziness. Even though she dropped out of treatment, she still completed both T1 and T2 measurements but did not want to complete the questionnaires in phase C anymore. The therapist recorded 5 DPICS scores from the CDI phase, with many weeks between sessions.

Participants 13 and 14: a married father and mother participated in treatment together with their 4-year-old girl. The father followed the treatment in Dutch and the mother in English due to her non-Dutch background. The father had a history of medical problems and substance abuse. The mother had psychological problems for which she started treatment during the PCIT trajectory. The child was clinically diagnosed with severe disruptive behavior, parent-child relationship problems, and parenting problems and there were suspicions of ASD. The child went to school 3 days a week upon request of the school due to hindrance from the disruptive behavior. They were referred to PCIT due to disruptive behavior at home and in school. The CDI phase was successfully completed (partially at home) and there were a few PDI sessions. The therapist recorded 5 DPICS scores from the CDI phase. The mother practiced with VR twice early on and said it helped her understand examples of how to use the skills. However, she later said it was not useful anymore. The father practiced once. Over the summer holidays, the child returned to their home country for 2 months, and PCIT was paused accordingly. In between, the parents were asked to practice with VR; however, they were unable to make time for this. PCIT was not restarted as they experienced enough improvement. Thus, they dropped out of treatment and the study. Due to drop out, only descriptive and visual inspection was included from this family. They were excluded from the pretreatment, posttreatment, and follow-up measurement analyses.

Participants 15 and 16: a married father and mother participated together in treatment with their 2-year-old girl. They received in-home PCIT. The parents experienced friction due to different views on parenting. The child had temper tantrums and exhibited crying fits and disruptive behavior. There were problems in the parent-child relationships. The reasons for referral were to lessen child disruptive behavior and parenting stress and the parents wished to improve their ability to react sensitively and responsively to their child. Upon completion of PCIT, they were positive about the results, although hesitant about some skills during the PDI phase. The father practiced a few times with VR but was not engaged in it. The mother was positive about practicing the skills in VR but found it difficult to make time for it. They both felt VR did not match their needs during the PDI phase. The therapist recorded 16 DPICS scores, all from the CDI phase.

Participants 17 and 18: a divorced father and mother participated together in treatment with their 7-year-old girl. The father was recovering from burnout and other psychological problems at the start of treatment. The mother had previously experienced burnout. The child was clinically diagnosed with ADHD, combined type, for which he received medication that was still being stabilized during the first few sessions of PCIT. There were parent-child relationship problems. Moreover, there were suspicions of oppositional defiance disorder and ASD. The child was referred for diagnostic assessment of oppositional defiance disorder and ASD and treatment for disruptive behavior. The parents originally did not want to participate in the study, but they did wish to practice with VR when their child was with the other parent. Upon completion of PCIT, the parents were positive about the results as the disruptive behavior decreased immensely, the child listened better, and parents were able to better understand their child. Moreover, both parents felt that the parent-child relationship had bettered during treatment. They mentioned that sudden changes or adaptations remained difficult for their child posttreatment. The father was positive about VR, although he experienced technological problems at first. The mother experienced nausea and dizziness when practicing in VR, so tried to do the videos on her phone without the head-mounted display headset. However, she did not feel it helped her and found the scenarios too simple. The parents were willing to participate in posttreatment and follow-up measurements and in phase C. The therapist recorded 11 DPICS scores, all from the CDI phase.

### Primary Measures

#### Visual Inspection Analyses: VR, DPICS, ECBI, and OBVL-K

Multimedia Appendix 2 shows the visual inspection analyses per participant. In all figures, the horizontal axis represents the time in treatment expressed in weeks and the vertical lines represent the separation between subsequent phases and the addition of VR. Specifically, the gray line separates the baseline and CDI phase, the blue line separates the CDI and PDI phases, the purple line separates the PDI and follow-up phases, and the red dotted line represents the addition of VR. The visual inspection analysis of VR is shown for all participants except for participants 10 and 11 as they did not want to receive the addition of VR any longer. However, when they would have received VR is vertically presented. The visual inspection analyses were only done for DPICS positive following and negative leading when DPICS scores were archived during treatment. This means that all participants except for participants 02, 03, 05, 06, and 09 were shown. We hypothesized that there would be a visible increase in positive following and a visible decrease in negative leading as soon as VR was introduced in treatment. For the ECBI and OBVL-K, visual inspection analyses were shown for all participants except for participant 14, as this participant did not fill out questionnaires due to the language barrier. We expected that the ECBI and OBVL-K would visually decrease over time, with a potential lag of 2 or 3 weeks behind the DPICS scores dependent on the respective increasing of positive following scores and decreasing negative leading scores.

Following visual inspection of the plots, little discernable change was apparent when looking at the moment VR was added to the treatment and the following DPICS scores. When examining the plots, participants 04, 17, and 18 appeared to show an increase in DPICS positive following scores when VR was added to treatment. Furthermore, participant 15 appeared to start using VR at around increment 10, and when looking at the DPICS positive following it appeared to increase at around the same time point. There was a visible upward trend in DPICS positive following (where everything >30 is considered good) for participants 04, 11, 12, 13, 14, 15, 16, 17, and 18. Participant 10 appeared not to show a steady pattern in DPICS positive following. There was also a visible downward trend in DPICS negative leading (where everything <5 is considered good) for participants 04, 10, 11, 12, 13, 14, 15, 16, and 18. No discernable change was visible for participant 17 in DPICS negative leading.

Following visual inspection of the plots for the ECBI, a visible decline in scores appeared for participants 01, 02, 03, 09, 12, 17, and 18. Participant 06 and 15 seemed to first decrease, but subsequently fluctuate in scores. There seemed no noteworthy changes in ECBI scores for participants 04, 05, 11, and 16. For participants 10 and 13, there appeared to be an increase in ECBI scores over time. No discernable change appeared between the use of VR and ECBI through visual inspection.

Following visual inspection of the plots for the OBVL-K, a visible decline in scores appeared for participants 01, 02, 06, 09, 12, 15, 17, and 18. The scores on the OBVL-K seemed to increase for participants 05, 10, and 13. There were no visible changes for participants 03, 04, 11, and 16 as scores seemed to remain stable or fluctuated a lot over time. No discernable change appeared between the use of VR and OBVL-K through visual inspection.

#### Randomization Tests: ECBI and OBVL-K

Table 2 shows the randomization tests of the ECBI Intensity Scale and the OBVL-K of all scores visible per participant in Multimedia Appendix 2. The effect size and individual *P* values are shown for both questionnaires. Only participants with at least 2 measurement points in phases A (baseline) and B (intervention) were included to assess treatment effects. This means that participants 17 and 18 were excluded as they did not participate in phase A.

The randomization test for the ECBI Intensity Scale revealed that there was no overall group significance in reports of child disruptive behavior for the entire group, nor solely for treatment completers. Only participant 09 showed a very large effect size complemented by a statistically significant decrease in reports of child disruptive behavior. Moreover, large or very large positive effect sizes were also found for participants 01, 02, 06, 11, 12, and 15, indicating that there were observed positive effects of their reports of child disruptive behavior, but these were not statistically significant. Nonetheless, this means that treatment positively impacted the reported levels of child disruptive behavior for a large number of participants. Participants 05 and 10 showed large to very large contratherapeutic effects in their reports of child disruptive behavior, although again not statistically significant. However, this means that for these 2 participants, their reports of child disruptive behavior were overall higher during the treatment phase than in the baseline phase, potentially due to amounting stressful circumstances described earlier.

The randomization test for the OBVL-K revealed that there were no overall group significance in reports of parenting stress. Again, participant 09 showed a very large effect size, complemented by a statistically significant decrease in parenting stress. Very large effect sizes were found for participants 06, 11, and 15, indicating that there were observed therapeutic effects of their reports of parenting stress, although these were not statistically significant. Nonetheless, this means that treatment positively impacted their reported levels of parenting stress. Participant 10 (who dropped out) demonstrated a nonsignificant very large contratherapeutic effect size in his reports of parenting stress, meaning that his parenting stress was higher during the treatment phase than in the baseline phase, potentially due to amounting stressful circumstances described earlier.

**Table 2:** Randomization tests per participant showing effect size and significance of the Eyberg Child Behavior Inventory (ECBI) Intensity Scale and the Dutch Parenting Stress Questionnaire, shortened version (Opvoedingsbelasting vragenlijst-Kort; OBVL-K).

Participant	ECBI Intensity Scale			OBVL-K	
	Cohen *d*	*P* value	Cohen *d*		*P* value
01^a^	3.59^b^	.84	3.59^c^		.82
02	0.80^d,e,f^	.20	0.69		.15
03	0.394	.68	0.48		.49
04	0.002	.50	0.06		.46
05	−0.925^d,e,f^	.92	−0.47		.81
06	1.44^c^	.24	1.60^c^		.14
09	1.36^c^	.001^g^	1.39^c^		.001^e^
10^a^	−2.17^b^	.10	−1.77^c^		.10
11^a^	1.74^c^	.18	1.76^c^		.07
12^h^	0.86^d,e,f^	.25	0.88^d,e,f^		.13
13^a^	0.20	.48	0.217		.43
15	2.04^b^	.27	2.04^b^		.20
16	0.49	.28	0.32		.32
Treatment completers	—^i^	.14	—		.14
Total	—	.27	—		.24

^a^Dropped out fully.

^b^Cohen *d* effect size: huge=2.00.

^c^Cohen *d* effect size: very large=1.20.

^d^Cohen *d* effect size*:*large=0.80.

^e^Cohen *d* effect size*:*medium=0.50.

^f^Cohen *d* effect size*:* very small=0.01 to small=0.20

^g^Significance at <.05.

^h^Dropped out of intervention but remained in study.

^i^Not applicable.

### Virtual Reality

To investigate whether there was a visible effect in the use of parenting skills from one PCIT session to the next when parents used VR, we subtracted the number of tallied skills in a session from its previous session. As parents need to achieve a preestablished amount of positive parent skills, including 10 labeled praises, 10 behavior descriptions, and 10 reflections, we corrected for 30 tallied skills. This means that any number >30, was considered the same. All participants who had practiced with VR between 2 PCIT sessions, and where DPICS scores during the 5-minute observation at the start of both sessions were available, are included in Table 3. This table reflects how practicing with VR in between sessions contributes to the positive following and negative leading scores of parents. As DPICS scores were archived from varying sessions and parents practiced at varying moments in time, this table does not provide information regarding specific sessions and the effects of VR on those sessions. It does consider the number of times practiced between 2 sessions. Both the mean and median are shown. The median is more sensitive to outliers, which made it a more cautious analysis.

Table 3 shows that when parents used VR, all their negative leading scores decreased from one session to the next, both when looking at the median difference and the mean difference. In addition, only for 2 parents, the positive following skills increased from one session to the next when VR was used. This suggests that when parents practiced with VR in between sessions, it especially led to a decreased use of negative leading statements in the following session.

**Table 3:** Median and mean difference in positive following and negative leading skills from one session to the next when virtual reality was used in between sessions^a^.

Participant	Median difference			Mean difference	
	Positive following	Negative leading	Positive following		Negative leading
04	7.00	0.00	3.00		−0.56
15	−14.50	−5.00	−9.00		−6.75
16	0.00	−2.00	−6.33		−0.33
17	0.00	−1.50	−1.13		−1.30
18	4.00	−2.00	4.00		−1.75

^a^Positive following is supposed to increase for wanted change to occur, and negative leading is supposed to decrease for wanted change to occur.

### Secondary Measures

#### DPICS Group Outcomes

As the sample was too small to perform *t* tests, a nonparametric test (the Wilcoxon signed rank test) was performed to illustrate the changes in positive following and negative leading during the CDI phase according to the DPICS from pretreatment to posttreatment and follow-up (Table 4). Effect sizes were demonstrated with Cohen *d* [57].

Significant effects were found both in positive following and in negative leading from pretreatment to posttreatment, and from pretreatment to follow-up. No significant effects were found from posttreatment to follow-up. There was a general increase in mean in the use of positive following skills and a general decrease in the use of negative leading statements. Moreover, the effect sizes according to Cohen *d* indicated very large effects from pretreatment to posttreatment and from pretreatment to follow-up for positive following, as well as very large effect size for pretreatment to follow-up in negative leading.

**Table 4:** Changes in positive following and negative leading during the child-directed interaction phase in pretreatment, posttreatment, and follow-up measurements for parents who completed Parent-Child Interaction Therapy^a^.

Measurements		Sample size, n	Wilcoxon signed rank test, mean (SD; range)	*P* value	Cohen *d*
**Positive following**					
	T0^b^	9	5.11 (5.64; 0-18)	T0 to T1^c^=.01^d^	T0 to T1=1.84^e^
	T1	10	32.10 (22.87; 11-89)	T0 to T2^f^=.04^d^	T0 to T2=2.96^e^
	T2	7	32.00 (13.49; 13-51)	T1 to T2=.40	T1 to T2=0.01
**Negative leading**					
	T0	9	30.44 (15.06; 5-56)	T0 to T1=.05^d^	T0 to T1=1.02^g,h,i^
	T1	10	14.40 (16.41; 0-57)	T0 to T2=.04^d^	T0 to T2=2.27^e^
	T2	7	6.43 (4.86; 1-15)	T1 to T2=.06	T1 to T2=0.68

^a^Positive following must increase and negative leading must decrease for wanted change to have occurred.

^b^T0: pretreatment measurement.

^c^T1: posttreatment measurement.

^d^Significance at <.05.

^e^Cohen *d* effect size: very large=1.20.

^f^T2: follow-up measurement.

^g^Cohen *d* effect size: large=0.80.

^h^Cohen *d* effect size: medium=0.50.

^i^Cohen *d* effect size: very small=0.01 to small=0.20.

#### VR, DPICS, and RCIs for ECBI and OBVL

To illustrate the number of times participants practiced with VR and whether this affected the primary outcomes of positive parenting skills, child disruptive behavior, and parenting stress, we examined the raw scores per person. Reliable change was not calculated for the DPICS, because there was not enough published information for the calculation of reliable change. Therefore, we have merely shown the raw scores of positive following and negative leading per measurement point. As this is a clinical sample, we deemed it important to show raw total scores and demonstrate whether or not participants fell into the clinical range at different time points (Multimedia Appendix 3; Table 5). To further illustrate changes in child disruptive behavior and parenting stress, we also performed RCIs (Table 6). We only included the participants who had completed ≥2 measurement points, which meant that participants who had dropped out of treatment and the study were not included (ie, participants 01, 10, 11, 13, and 14).

To assess whether there were differences on the DPICS, ECBI, and OBVL scores when VR was practiced, we split the group in 2 based on the median use of VR in the group. The median of practicing with VR was 6. Therefore, we split the group into participants who practiced with VR 6 or less times (group A) and who practiced with VR 7 or more times (group B; Tables 5 and 6). With this, we provided preliminary observations regarding practice time and treatment outcomes, but more evidence is necessary to draw any conclusions on relations with one another, as other factors may also have been of influence.

First, we observed that 3 of the 5 (60%) participants from group A did not participate in the follow-up measurement, whereas all participants from group B participated in posttreatment and follow-up. Second, results show that group A started with higher scores in positive following and negative leading than group B. Group A consistently scored higher in positive following than group B, but only group B showed progression in positive following from posttreatment to follow-up. However, for negative leading, group B showed lower scores on pretreatment and posttreatment than group A. Both groups decreased in negative leading from posttreatment to follow-up. Third, group A also started with higher reports on the ECBI Intensity Scale than group B and only group B had all scores less than the clinical cutoff score at posttreatment and at follow-up. Fourth, although group B started with a higher overall score on the ECBI Problem Scale, they diminished more than group B and remained below the clinical cutoff score at posttreatment and follow-up. Fifth, group A showed higher reports of total parenting stress in all 3 measurement points than group B. Overall, we observed more improvement for group B than for group A in child disruptive behavior and parenting stress, but group A applied more parenting skills than group B.

These results show that there are little discernable differences in terms of reliable change for the ECBI scales when splitting the group in 2 based on the median VR use. A total of 4 of 11 (36%) parents experienced a reliable change from pretreatment to posttreatment on the ECBI Intensity Scale. Two parents (both who practiced with VR >6 times) maintained that significant change at follow-up as well. For the ECBI Problem Scale, we observed that there were 6 (55%) parents with a reliable change from pretreatment to follow-up. Participant 16 experienced an increase in problems, which indicated contratherapeutic treatment effects. We were privy to the information that the child had experienced impetigo and chicken pox in the weeks before filling out the questionnaires, thus elevating her disruptive behavior, which could have influenced parent’s reports. Participant 06 also experienced a deterioration from posttreatment to follow-up. Participants 02, 09, and 12 had reliable changes from pretreatment to follow-up and participant 02 also showed a large reliable change from posttreatment to follow-up, indicating that the treatment seemed to continue its effects.

For the OBVL, we observed that in the group that practiced with VR <6 times, all parents experienced a large significant change in parenting stress from pretreatment to follow-up. Participant 06 showed a significant negative reliable change from posttreatment to follow-up, indicating deterioration. This is in line with the fact that parents were unable to make time for the observation at follow-up, due to increased psychological burden at that moment in time. Her partner, participant 05, although not significantly changed, reported even higher levels of parenting stress at follow-up than participant 06. In the group that practiced with VR >6 times, all participants showed a reliable change from pretreatment to follow-up, except participant 04 who experienced contratherapeutic treatment effects. As mentioned before, participant 04 started working full time at the end of treatment and expressed feeling parenting stress as a result. Although participant 18 showed a negative reliable change from posttreatment to follow-up, the parent gave us relevant information that could have influenced the change index. Namely, in the specific week that the questionnaires were filled out, 3 family members had celebrated their birthdays, which meant that the normal routine had been disturbed. The parent expressed that her child was tired and irritable as a result, which in her eyes, was not representative of the months posttreatment.

**Table 5:** The total number of times practiced with virtual reality (VR) and the raw scores in pretreatment, posttreatment, and follow-up measurements in the Dyadic Parent-Child Coding System (DPICS), Eyberg Child Behavior Inventory (ECBI), and Opvoedingsbelasting vragenlijst (OBVL) per participant.

Participant			VR		DPICS															ECBI															OBVL				
			Total		Positive following^a^							Negative leading^b^							Intensity scale								Problem scale							Total parenting stress					
					T0^c^		T1^d^		T2^e^		T0			T1		T2		T0				T1		T2		T0			T1		T2		T0				T1		T2
**VR practice group A (practiced with VR ≤6 times)**																																							
	03	4		3		89		—^f^		14			0		—		140				143		132		17			20		21		72				68		66	
	05	3		10		23		—		41			57		—		159				161		131		18			22		5^g^		79				79		75	
	06	3		6		28		—		36			19		—		155				109^g^		150		16			1^g^		14		75				55^g^		66	
	15	4		0		11		36		32			12		8		165				115^g^		129^g^		20			0^g^		0^g^		63				53^g^		48^g^	
	16	5		18		51		37		56			10		1		137				124^g^		135		15			1^g^		20		77				76		72	
**VR practice group B (practiced with VR ≥7 times)**																																							
	02	7		2		24		51		5			3		3		117^g^				63^g^		62^g^		29			20		0^g^		56^g^				54^g^		48^g^	
	04	24		2		17		24		30			8		10		123^g^				119^g^		131		13			17		18		72				72		76	
	09	14		2		35		43		37			22		15		145				128^g^		128^g^		21			7^g^		2^g^		70				68		66	
	12^h^	7		3		3		1		51			18		18		164				88^g^		87^g^		19			6^g^		7^g^		72				53^g^		51^g^	
	17	24		—		26		13		—			6		4		—				52^g^		51^g^		—			0^g^		0^g^		—				58^g^		50^g^	
	18	8		—		17		20		—			7		4		—				78^g^		101^g^		—			0^g^		2^g^		—				49^g^		59^g^	

^a^Positive following must increase.

^b^Negative leading must decrease.

^c^T0: pretreatment.

^d^T1: posttreatment.

^e^T2: follow-up.

^f^Not applicable.

^g^Raw scores below the clinical range (ECBI Intensity Scale <60; OBVL total T score <60).

^h^Dropped out of intervention but remained in study.

**Table 6:** Reliable change indices for the Eyberg Child Behavior Inventory (ECBI) and the Opvoedingsbelastingvragenlijst (OBVL) from pretreatment to posttreatment and to follow-up per participant.

Participants		ECBI Intensity Scale				ECBI Problem Scale				OBVL Total Parenting Stress		
		T0^a^ to T1^b^	T0 to T2^c^	T1 to T2	T0 to T1		T0 to T2	T1 to T2	T0 to T1		T0 to T2	T1 to T2
**VR^d^** **practice group A (practiced with VR ≤6 times)**												
	03	−0.14	0.37	0.50	−0.64		−0.85	−0.21	2.44^e^		3.66^e^	1.22
	05	−0.09	1.29	1.38	−0.85		2.77^e^	3.62^e^	0.00		2.38^e^	2.38^e^
	06	2.11^e^	0.23	−1.88	3.20^e^		0.43	−2.77^e^	11.90^e^		5.36^e^	−6.55^d^
	15	2.29^e^	7.57^e^	5.28^e^	4.26^e^		4.26^e^	0.00	5.95^e^		8.93^e^	2.98^e^
	16	0.60	0.09	−0.50	−0.43		−25.57^e^	−25.14^e^	0.60		2.98^e^	2.38^e^
**VR practice group B (practiced with VR ≥7 times)**												
	02	2.48^e^	2.52^e^	0.05	1.92		6.18^e^	4.26^e^	1.22		4.88^e^	3.66^e^
	04	0.18	−0.37	−0.55	−0.85		−1.07	−0.21	0.00		−2.44^e^	−2.44^e^
	09	0.78	0.78	0.00	2.98^e^		4.05^e^	1.07	1.22		2.44^e^	1.22
	12	3.49^e^	3.53^e^	0.05	2.77^e^		2.56^e^	−0.21	11.59^e^		12.80^e^	1.22
	17	—^f^	—	0.05	—		—	0.00	—		—	4.88^e^
	18	—	—	−1.06	—		—	0.43	—		—	−6.10^e^

^a^T0: pretreatment.

^b^T1: posttreatment.

^c^T2: follow-up.

^d^VR: virtual reality.

^e^Significant reliable change indices (RCI) values (ECBI RCI ≥1.96 or RCI ≤−1.96; OBVL RCI ≥1.645 and RCI ≤−1.645).

^f^Not available.

## Discussion

### Principal Findings

We experimentally examined the staggered addition of VR to PCIT with parents who followed treatment with their child who displayed child disruptive behavior. Our findings are presented in an SCED study on PCIT-VR in a community-based clinical sample. As this was the first time that VR was used supplementary to PCIT, we sought to give initial insights into the added value of this skill practice tool, and we examined the effects of additional practice time in VR and other treatment-related outcomes. Accordingly, we expected that the introduction of VR would increase positive parenting skills at a faster pace, and that this increase would subsequently lead to a faster diminishment of child disruptive behavior and parenting stress. This was partially confirmed as the relationship between practicing in VR and treatment-related outcomes varied among participants. Some participants seemed to benefit from the addition of VR, whereas it did not seem to impact treatment-related outcomes for other participants. We discuss the most important individual and group findings subsequently, after which we review strengths and limitations of the study and suggest future directions for research. Finally, we discuss clinical implications and conclude our findings.

With regards to the visual inspection plots and randomization tests, we found that overall, there did not seem to be a clear relationship between the use of the VR tool and PCIT treatment effects, as positive parenting skills did not noticeably change when VR was added in most of the cases. However, in 3 cases, positive parenting skills visibly seemed to increase when VR was introduced to treatment. This was confirmed through median and mean differences in skills between 2 sessions. Specifically, our study demonstrated that there seemed to be meaningful differences for more parents in changes in negative leading statements (ie, questions, commands, and negative talk) than in positive parenting skills (eg, labeled praises, reflective statements, and behavioral descriptions). Although our VR tool specifically targeted scenarios aimed at practicing positive parenting skills, some scenarios also targeted practicing ignoring unwanted behavior, while using positive parenting skills to re-engage the child in play. Although there is no clear reasoning behind why practicing in VR seemed to be associated more with decreased questions and commands in the following session, we can venture a guess as to why this pattern emerged. When parenting skills were sequenced, visible changes in increased positive parenting skills and decreased negative leading statements were seen during treatment sessions in a study by Hakman et al [58]. That study attributed this change to therapists providing immediate feedback to the parent to appropriately respond to child behaviors, and the therapist being able to tailor the application of skills when problems arose during sessions. That described mechanism of feedback and tailoring responses is incorporated into our VR tool. Parents receive immediate positive feedback for correctly choosing to use positive skills and receive suggestions for alternative skill application when choosing to use questions and commands. Therefore, although they may not yet be fully proficient in applying positive parenting skills, practicing in VR may have given them enough alternatives to negative leading statements to limit those in the following sessions. While this was an incidental finding, we deem it an important benefit that pleads for practicing in VR as we know that negative parenting strategies can increase or maintain child disruptive behavior and other adverse outcomes [59].

Considering the number of missing questionnaires from parents and the amount of times VR was practiced with, our results indicate that the effect of VR may potentially be dependent on the level of engagement from parents in treatment. Parents who filled out >70% of questionnaires and did not drop out of either the treatment or the study, seemed to practice with VR more. This insinuates that when families have the drive, motivation, and capacity to change, in addition to having the mental space to take on the responsibility of change, they seem to take accountability for that change to happen. In other words, when they feel accountable for change, they do everything in their power, including increasing their practice time through VR, for the desired change to happen. This finding emphasizes treatment engagement of parents, which is considered an important predictor for treatment success [60]. In line with our study, previous research showed that lower income and high levels of parental stress were associated with lower treatment engagement [61,62]. Moreover, the more engaged parents were in treatment, the lower the child’s disruptive behavior was at posttreatment and the more parents expressed understanding skills and how to use them appropriately [61]. In addition, Pocket PCIT found that treatment engagement was stimulated through their web-based resource platform for parents and they suggested this as a point of interest for future research [24]. Although we attempted to enhance treatment engagement through practicing with skills in VR, it may be that when the psychological burden is too high, parents are unable to benefit enough from the additional services offered to them. Another possibility is that due to the use of 360-degree videos and thus creating a 3-degree of freedom virtual environment, VR may not have been engaging enough for some participants who had previous experiences with VR. Where VR could have been perceived as an engaging way to practice skills, the static storyline could have quickly led to boredom [63]. This, in turn, could have led to less practice time in the virtual environment. Taken together, we believe that the VR practice could potentially work best as a suggested additional practice tool tailored to the needs, capacity, and motivation of individuals, rather than a mandatory addition to treatment.

We did not uncover conclusive changes between practicing with VR and child disruptive behavior, nor between practicing with VR and parenting stress, although therapeutic trends are visible in parenting skills, child disruptive behavior, and parenting stress. These were complemented by large effect sizes after receiving PCIT in most (n/N, %) cases. One parent, who had dropped out before treatment completion, showed contra therapeutic treatment-related outcomes, where both child disruptive behavior and parenting stress increased over time. These individual changes were also confirmed in the pretreatment, posttreatment, and follow-up measurements. Here, we found significant changes in increased positive parenting skills and decreased questions and commands from pretreatment to posttreatment as well as from pretreatment to follow-up for treatment completers. These were again complemented by very large effect sizes for receiving PCIT (-VR) in general. Our findings are in line with treatment effects found in other PCIT studies, including findings from a study in a similar Dutch community-based clinical sample [1,13,64]. This suggests that, regardless of the addition of VR, PCIT had the wanted therapeutic effects on the children and parents from this community-based clinical sample.

Furthermore, reliable decreases were found in parenting stress for all but 1 of the 11 (91%) treatment completers from pretreatment to follow-up, regardless of the number of times they had practiced with VR. This means that parenting stress decreased for all parents in our sample because of following PCIT (-VR). There was variability in reliable decreases of child disruptive behavior from pretreatment to posttreatment and to follow-up, where 5 (45%) parents reported reliable decreases from pretreatment to follow-up, and 1 (9%) parent reported a reliable increase, which was contra-therapeutic. Moreover, the participants who practiced with VR >6 times, reported their child’s disruptive behavior to be below the clinical range at posttreatment, and all but 1 (83%) participant (who scored 131) remained below the clinical range at follow-up. This means that PCIT positively affected child disruptive behavior, but when VR was practiced along with it more than 6 times, the child disruptive behavior remained manageable at follow-up. These results were in line with treatment protocol because when positive parenting skills are correctly implemented, treatment-related outcomes such as child disruptive behavior should also decrease [11]. Although no group comparisons were statistically made and we must be very careful with this interpretation, participants that practiced with VR <6 times had higher initial reports of child disruptive behavior and parenting stress, and remained higher at posttreatment and follow-up, which could suggest that these participants represented more complex problems. When there are more complex problems, families in community-based samples are known to have lower homework completion rates, which is postulated to be related to barriers such as high levels of stress and busy schedules [19,65]. This confirms the trends seen in the individual outcomes, where treatment engagement may be contingent on the complexity of problems and their (mental) capacity. These findings suggest that the VR practice tool may not be the solution to engage these specific families in PCIT. This group might, for example, benefit more from other types of additional care, such as financial aid, to reduce life stressors. Moreover, more research is warranted to confirm the speculatory relationship between the complexity of problems and less practice time in VR.

Furthermore, we can see that skill acquisition largely increased at posttreatment and follow-up when VR was practiced >6 times. This is in line with the deliberate practice theory, which states that additional practice time can contribute to increased skill learning [66-68]. However, we cannot attribute this solely to practicing in VR, as parents who practiced <6 times with VR equally showed large increases in skills at posttreatment and follow-up. Moreover, PCIT is already known to stimulate increases in skill acquisition [1]. It is possible that the increased practice time in VR led to increased confidence in parenting in general, which was suggested by qualitative findings in PCIT-VR, which was a part of the study but published as a separate qualitative paper [63]. This implies that practicing parenting skills and receiving feedback in VR may have been beneficial for the confidence in skill application of these parents, which was reflected in showing more lasting treatment outcomes than the participants that practiced less with VR. Consequently, we believe that tailoring VR to the needs of parents could be of clinical relevance in PCIT because some parents from our sample visibly benefited from the additional practice time.

### Strengths, Limitations, and Future Directions

This study allowed us to take an in-depth look at the varying change trajectories in the treatment-related outcomes in PCIT, while also investigating the novel VR practice tool. We uncovered interesting trends that demonstrated the complexity of a community-based clinical sample. Moreover, through VR, split families received an opportunity to practice in-treatment taught skills without the presence of their child. In our sample, we had 4 (participants for whom this was relevant and who profited from this. This was facilitated by the strong design that an SCED provides, as we could individually follow both parents and report their progress separately, but simultaneously. In addition, the simplicity of our VR tool with 360 degrees videos readily available on a personal smartphone meant that it was very accessible and easy to use. These points highlight the potential of VR to be tailored to an individual’s needs, and that it could be implemented as an optional skill strengthener when necessary.

Although this study provides valuable initial insights into the added value of VR to PCIT, there are some limitations that must be addressed. We must first acknowledge that because of using an SCED design, parents were required to fill out weekly questionnaires at home, in their own time, rather than in the PCIT room before the start of treatment. Although this meant that we had data about child disruptive behavior and parenting stress from weeks that sessions did not take place, it also meant that this could be seen as additional homework by parents. In other words, on top of giving them additional practice time through VR, they may have felt that the weekly SCED questionnaires were an additional task or burden as well. This could potentially have influenced their practice time, because if they were short on time, they may have picked questionnaires over VR practice time. Moreover, we know that homework completion rates are already lower in community-based clinical samples [19,65], so adding questionnaires as a task may have impacted practice time, albeit live or in VR.

Another limitation was that a lot of DPICS data were missing in our sample due to archiving problems. Therefore, although we can very carefully draw conclusions about the effects practicing in VR had on treatment, we cannot say that it was the case for our entire sample, as we do not know. As mentioned, there seemed to be a trend that indicated that the more dedicated parents were to fill out questionnaires, which was translated into fewer missed values, the more they seemed to practice with VR. However, we missed DPICS data from some of these participants too (eg, participants 02 and 09). It would have been interesting to be able to report their change in positive following skills and negative leading behavior alongside VR as well. In the future, a better system for archiving DPICS data should be implemented.

Finally, this study had originally planned to evaluate the staggered addition of VR at the start of treatment, after 3 sessions or after 6 sessions. While the idea of such a design was strong, as we would be able to attribute changes in treatment outcomes on VR and not because of maturation, we were unable to include enough participants to be able to say anything on this in this study. Nonetheless, qualitative findings of PCIT-VR suggested that parents found particular added value in using VR at the start of treatment, as it provided them with a visual learning opportunity that complimented the verbal instructions from therapists. It also gave them examples of how to use specific skills, which they found helpful to apply to special time with their child [63]. With this knowledge, we believe in the potential VR has as a complementary tool to the instruction session. Therefore, it would be valuable to offer VR from the start for parents. A follow-up study with a similar design where DPICS scores are archived could help us confirm the initial suggestions that VR could help improve treatment outcomes.

### Conclusions

First, our study provides additional evidence that PCIT in a community-based clinical sample results in positive treatment effects. Moreover, we gave valuable first insights into the added value of VR to PCIT. Although this study alone does not provide strong enough evidence to clearly state that there was added value of VR to PCIT, we believe that there are interesting trends that plead for a further investigation into PCIT-VR. These findings tentatively suggest that practicing with VR could potentially increase positive parenting skills, and it could potentially also have an impact on other treatment-related outcomes, such as child disruptive behavior and parenting stress. However, individual differences, such as treatment engagement, accountability, capacity for change, and complexity of problems, may play a role both in the number of times VR was practiced with over the course of the treatment and on individual treatment effects. In other words, creating a practical add-on for such a clinical target population is beneficial if it fits their respective complex and stressful lives, as this increases the chances of them being (able to be) engaged, and thus achieving treatment success. Overall, we can preliminarily suggest that VR could be of added value to PCIT to increase confidence in parenting skills for certain parents, while simultaneously considering complex factors that play into treatment success.

## Appendix

Multimedia Appendix 1 Number of measurements completed and missing per participant per phase.

Multimedia Appendix 2 Visual inspection analysis for all participants.

Multimedia Appendix 3 The total number of times practiced with virtual reality and the raw scores in pretreatment, posttreatment, and follow-up measurements in the Dyadic Parent-Child Interaction Coding System, Eyberg Child Behavior Inventory, and the Dutch Parenting Stress Questionnaire per participant.

## Abbreviations

ADHD: attention-deficit/hyperactivity disorder
ASD: autism spectrum disorder
BPT: behavioral parent training
CDI: child-directed interaction
DPICS: Dyadic Parent-Child Interaction Coding System
ECBI: Eyberg Child Behavior Inventory
OBVL: Dutch Parenting Stress Questionnaire (Opvoedingsbelastingvragenlijst)
PCIT: Parent-Child Interaction Therapy
PDI: parent-directed interaction
RCI: reliable change index
SCED: single-case experimental design
SCRIBE: Single-Case Reporting Guideline in Behavioral Interventions
VR: virtual reality

## Data Availability

The datasets generated and analyzed during this study are not publicly available to protect the privacy of the participants and follow ethical guidelines, but they are available from the corresponding author on reasonable request.
