# Supplementary material for: The Added Value of Parents Practicing in Virtual Reality to Illustrate the Use of Innovative Methods in Parent-Child Interaction Therapy: Single-Case Experimental Design
Source: JMIR Pediatr Parent. 2025 Jul 23;8:e60752. doi: 10.2196/60752 (PMC12329383; doi:10.2196/60752)
Supplement: Multimedia Appendix 1 [file pediatrics_v8i1e60752_app1.docx]

Table 2

Number of measurements completed and missing per participant per phase.

| Participant (pp) | Randomization | Baseline  (phase A) | | T0  (pretreatment) | | Phase B  (PCIT) | | Phase B’  (PCIT with VR) | | Treatment (phase B & B’) | T1  (posttreatment) | | T2  (6-months follow-up) | | Phase C (follow-up) | | Total |
| --- | --- | --- | --- | --- | --- | --- | --- | --- | --- | --- | --- | --- | --- | --- | --- | --- | --- |
|  | Baseline \| VR | Completed | Missing | Questionnaires | DPICS | Completed | Missing | Completed | Missing | Sessions or months | Questionnaires | DPICS | Questionnaires | DPICS | Completed | Missing | % Completed |
| 01^1^ | 4 weeks \|  at the start | 4 | 0 | ✓ | ✓ | N/A | N/A | 8 | 3 | 1 session | - | - | - | - | - | - | 80 |
| 02 | 4 weeks \|  3 sessions | 4 | 0 | ✓ | ✓ | 5 | 0 | 29 | 6 | 08/20-04/22 | ✓ | ✓ | ✓ | ✓ | 2 | 1 | 85 |
| 03 |  | 4 | 0 | ✓ | ✓ | 4 | 1 | 13 | 22 |  | ✓ | ✓ | ✓ | - | - | - | 48 |
| 04 | 6 weeks \|  at the start | 6 | 0 | ✓ | ✓ | N/A | N/A | 28 | 0 | 18 sessions | ✓ | ✓ | ✓ | ✓ | 3 | 0 | 100 |
| 05 | 6 weeks \|  3 sessions | 5 | 2 | ✓ | ✓ | 2 | 1 | 10 | 14 | 08/21-03/22 | ✓ | ✓ | ✓ | - | 0 | 3 | 46 |
| 06 |  | 6 | 1 | ✓ | ✓ | 2 | 1 | 12 | 12 |  | ✓ | ✓ | ✓ | - | 1 | 2 | 57 |
| 09 | 5 weeks \|  3 sessions | 18 | 3 | ✓ | ✓ | 7 | 0 | 23 | 5 | 02/22-08/22 | ✓ | ✓ | ✓ | ✓ | 2 | 1 | 85 |
| 10^1^ | 4 weeks \|  6 sessions^4^ | 3 | 1 | ✓ | ✓ | 36 | 11 | N/A | N/A | 12 sessions | - | - | - | - | - | - | 76 |
| 11^1^ |  | 3 | 1 | ✓ | ✓ | 39 | 8 | N/A | N/A |  | - | - | - | - | - | - | 82 |
| 12^2^ | 5 weeks \|  at the start | 4 | 1 | ✓ | ✓ | N/A | N/A | 18 | 25 | 5 sessions | ✓ | ✓ | ✓ | ✓ | - | - | 46 |
| 13^1^ | 6 weeks \|  3 sessions | 2 | 5 | ✓ | ✓ | 2 | 1 | 22 | 15 | 15 sessions | - | - | - | - | - | - | 55 |
| 14^1,5^ |  | N/A | N/A | N/A | ✓ | N/A | N/A | N/A | N/A |  | - | N/A | - | N/A | - | - | N/A |
| 15 | 5 weeks \|  at the start | 3 | 2 | ✓ | ✓ | N/A | N/A | 23 | 17 | 35 sessions | ✓ | ✓ | ✓ | ✓ |  |  | 60 |
| 16 |  | 5 | 0 | ✓ | ✓ | N/A | N/A | 29 | 11 |  | ✓ | ✓ | ✓ | ✓ |  |  | 77 |
| 17^3^ | N/A | N/A | N/A | N/A | N/A | N/A | N/A | 13 | 6 | 08/21-05/22 | ✓ | ✓ | ✓ | ✓ | 3 | 0 | 73 |
| 18^3^ | N/A | N/A | N/A | N/A | N/A | N/A | N/A | 15 | 3 |  | ✓ | ✓ | ✓ | ✓ | 3 | 0 | 86 |

Note. Checkmarks (✓) indicate completion and numbers indicate the number of completed measurements. ^1^Dropped out fully; ^2^Dropped out of intervention but remained in study; ^3^Received VR with intervention and were monitored but due to being on a waitlist for a long time and wanting to start the intervention as soon as possible, they were not randomized and no baseline measurements were obtained; ^4^When given explanation, did not want to use VR due to added psychological burden; ^5^Participant did not fill out questionnaires as these were in Dutch and her Dutch level of reading/understanding was not high enough to complete questionnaires in Dutch.
