# Supplementary material for: The Added Value of Parents Practicing in Virtual Reality to Illustrate the Use of Innovative Methods in Parent-Child Interaction Therapy: Single-Case Experimental Design
Source: JMIR Pediatr Parent. 2025 Jul 23;8:e60752. doi: 10.2196/60752 (PMC12329383; doi:10.2196/60752)
Supplement: Multimedia Appendix 2 [file pediatrics_v8i1e60752_app2.docx]

Multimedia appendix 2.

Visual Inspection Analysis for all participants

Legend: In all figures, the horizontal axis is the time in treatment expressed in weeks, and the vertical lines represent the separation between subsequent phases and the addition of VR. More specifically, the gray line separates the baseline and CDI phase, the blue line separates the CDI and PDI phase, the purple line separates the PDI phase and follow-up phase, and the red dotted line represents the addition of VR.

*Participant 01
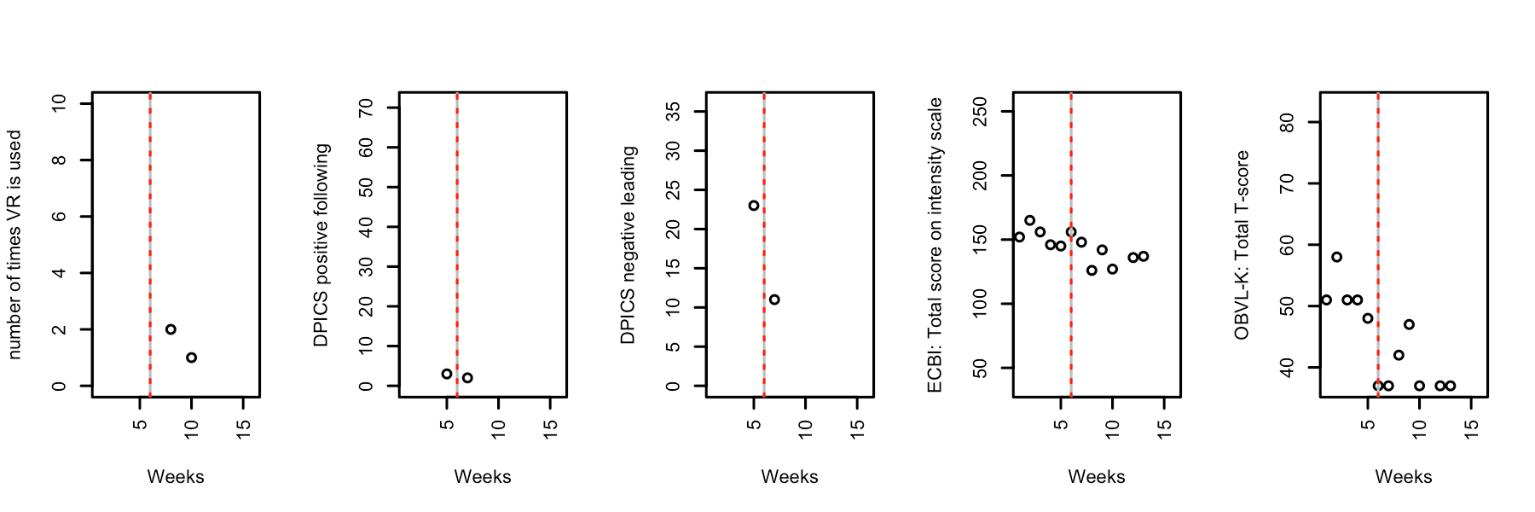
*

*Participant 02*

*
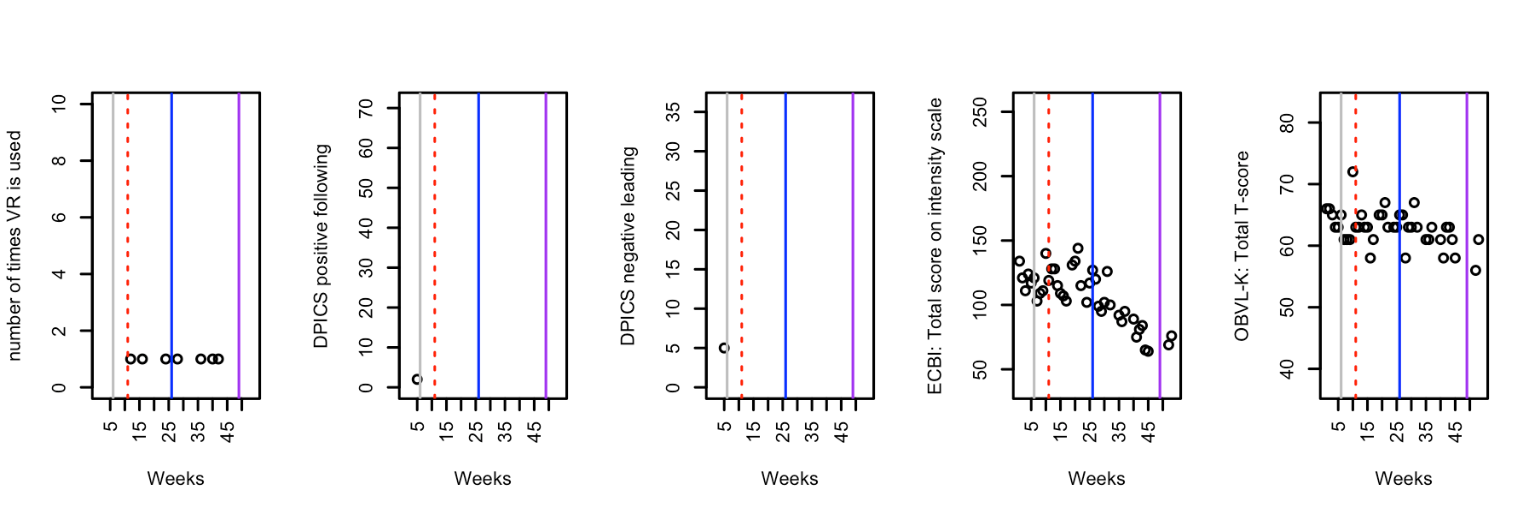

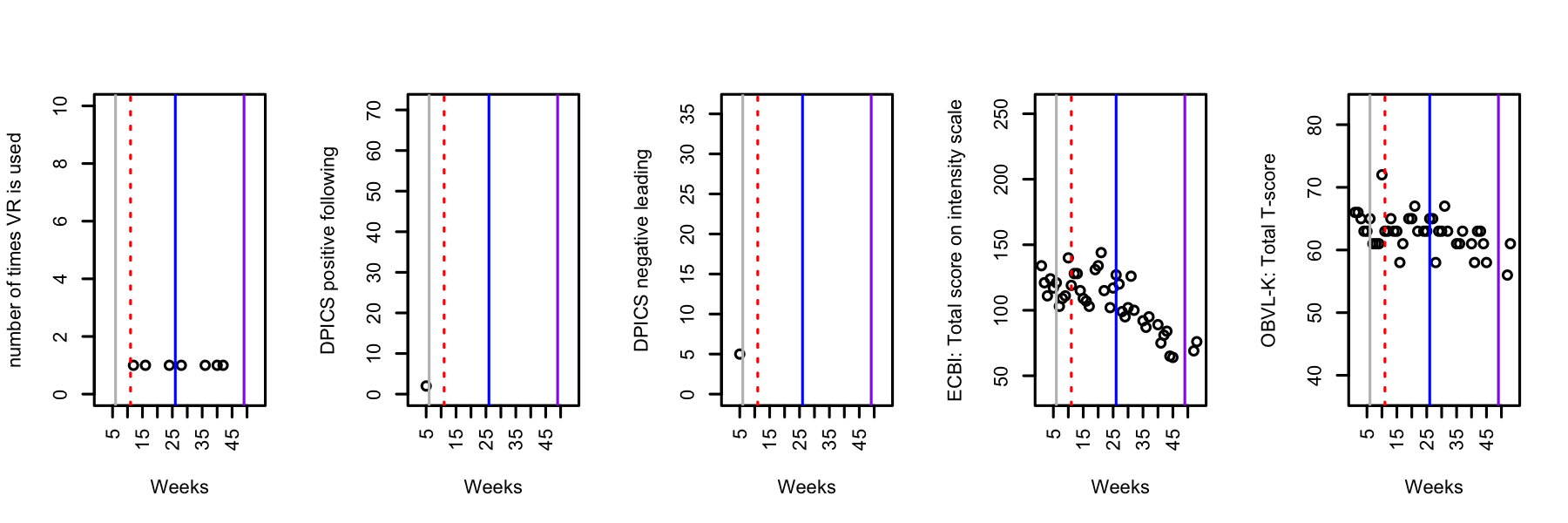
*

*Participant 03*

*
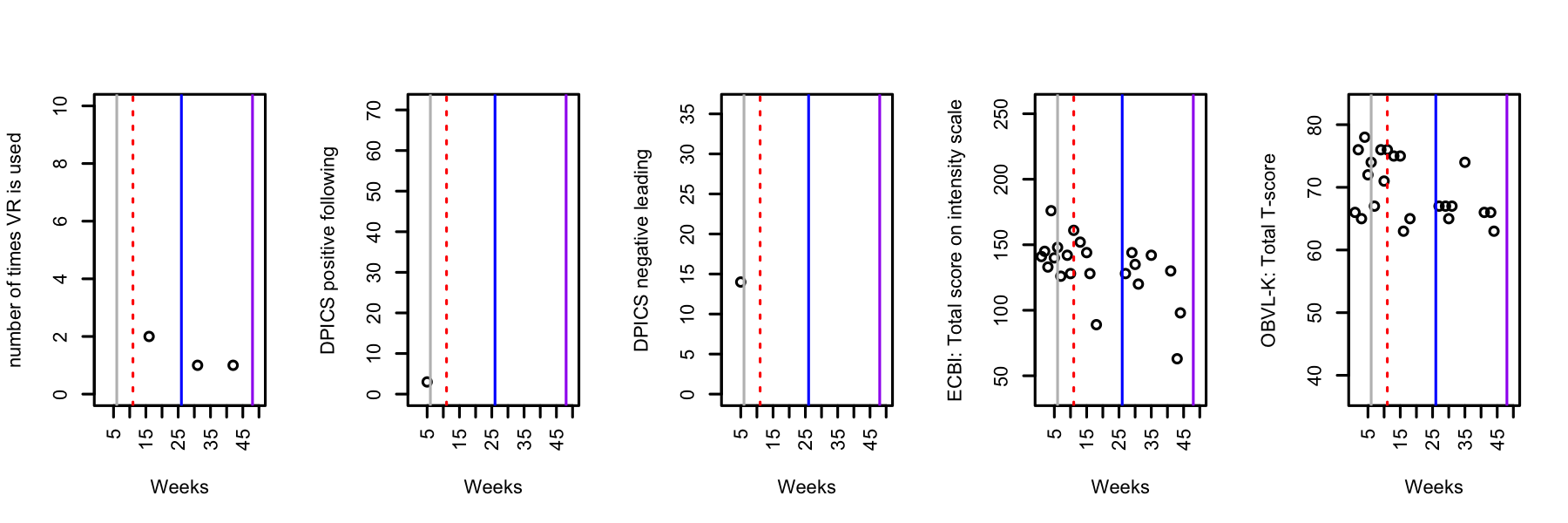

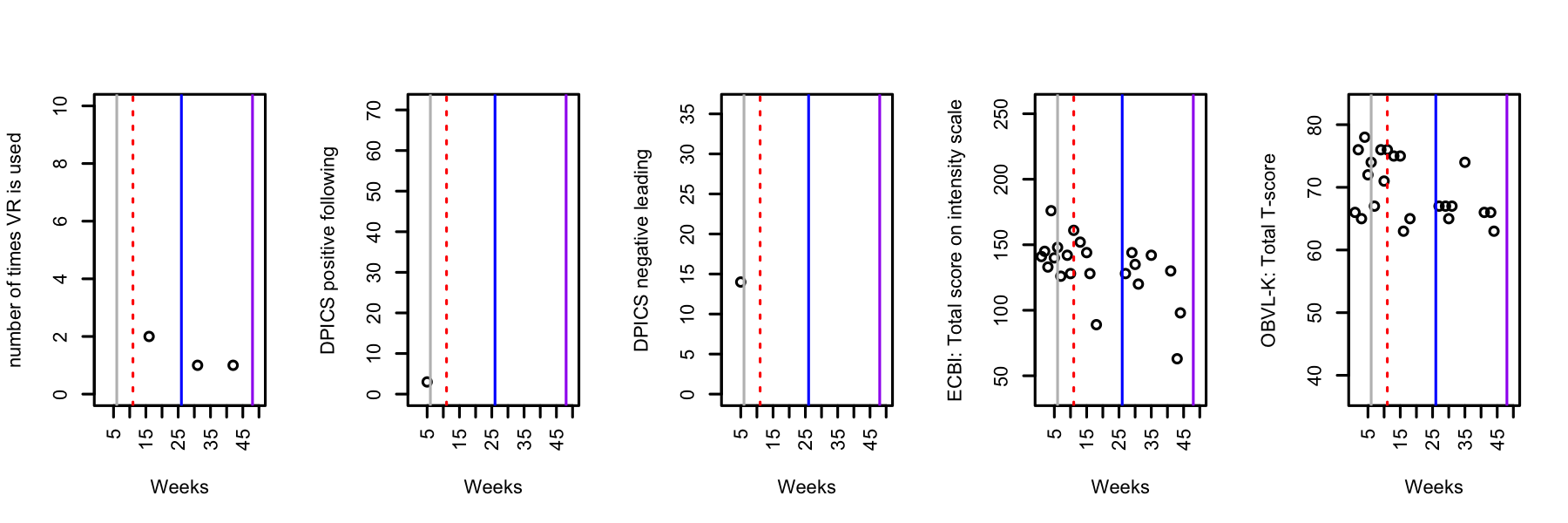
*

*Participant 04*

*
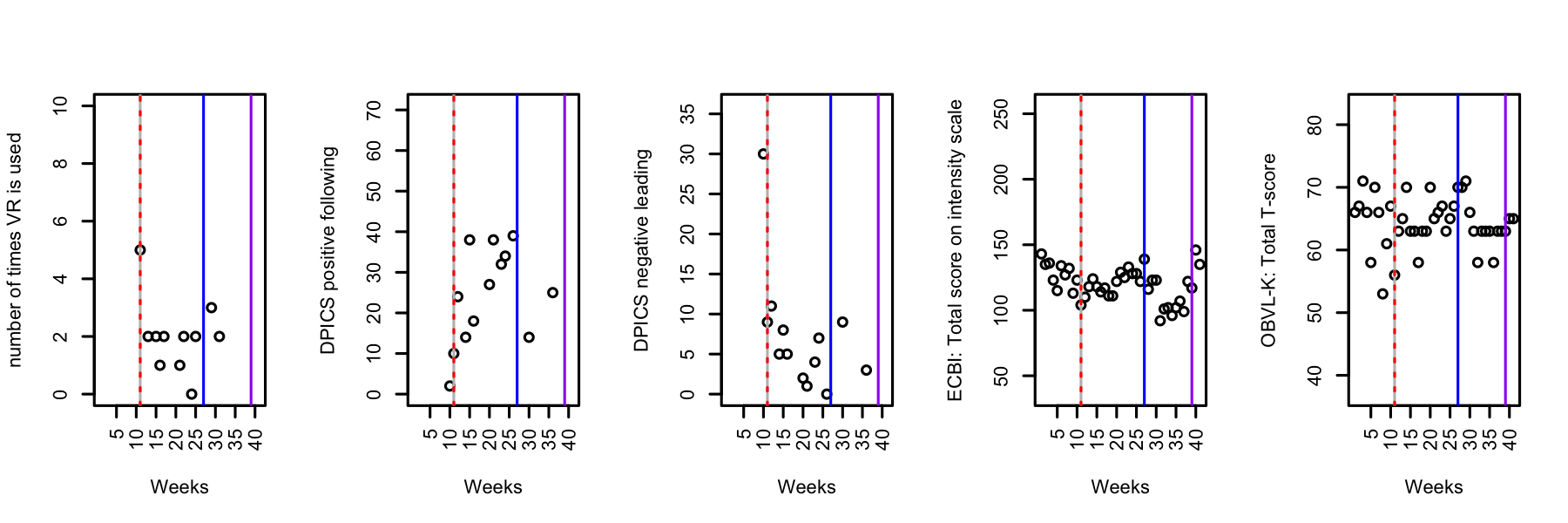
*

*Participant 05*

*
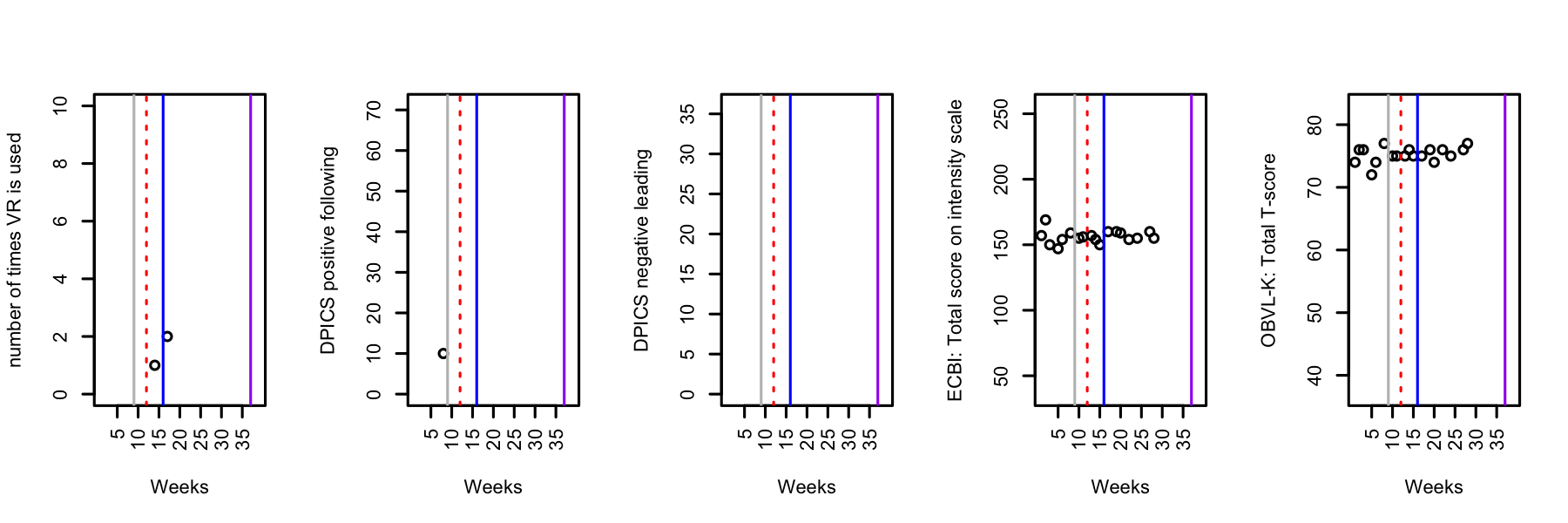

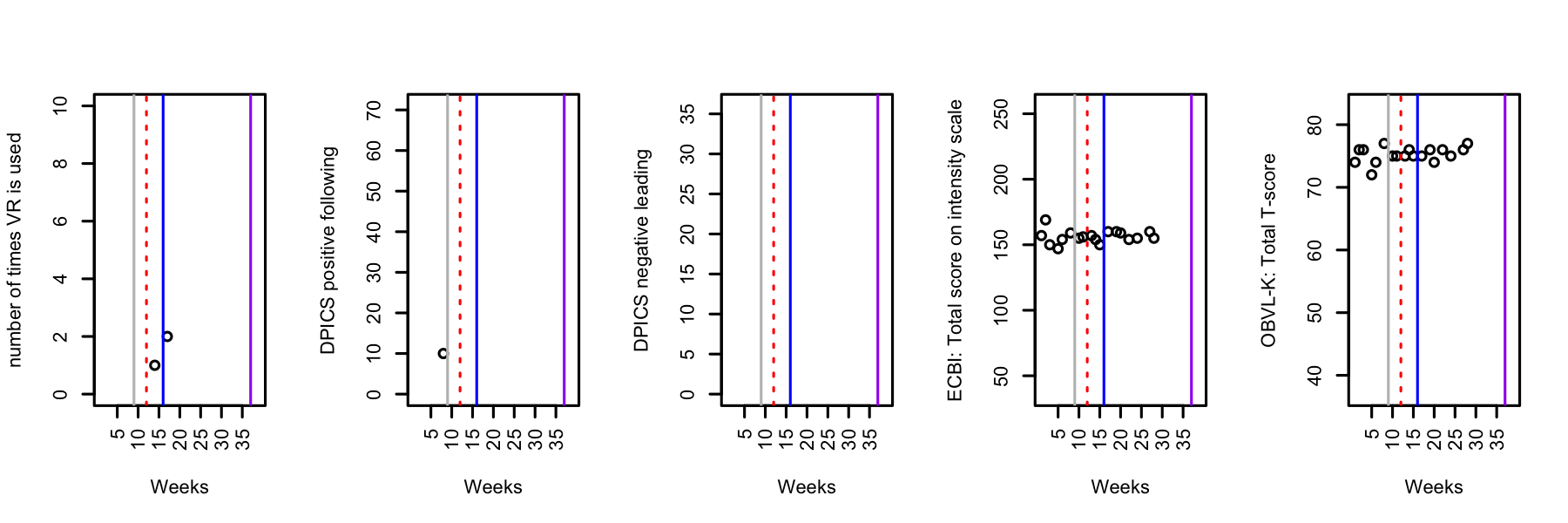
*

*Participant 06*

*
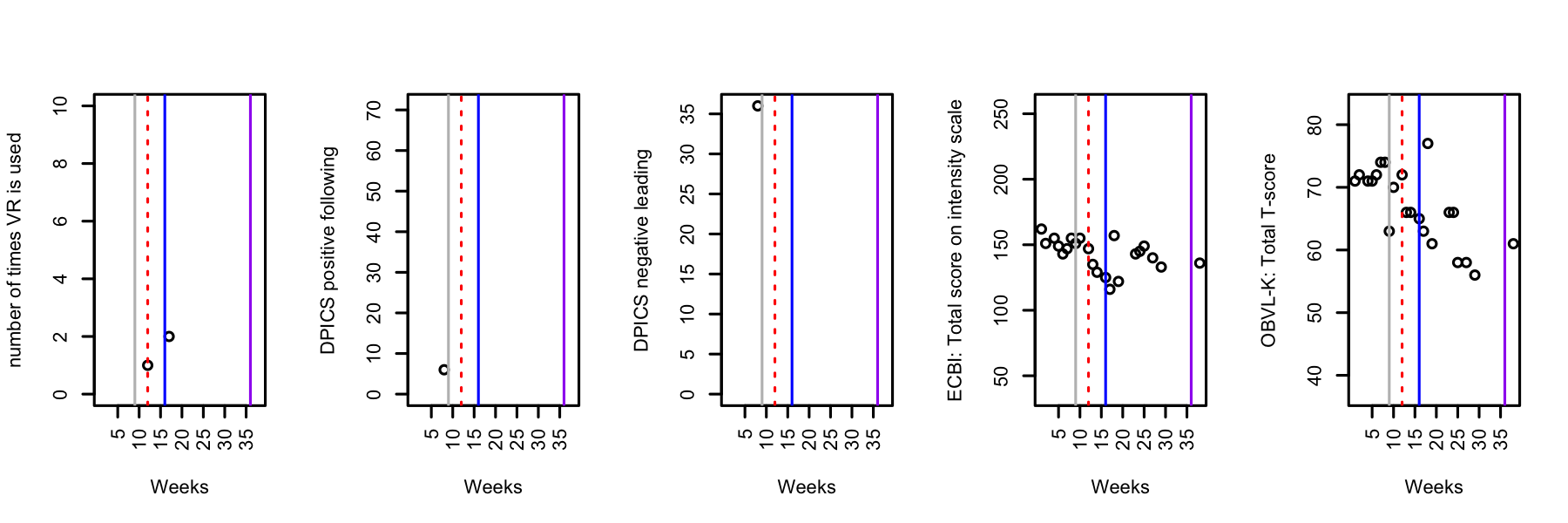

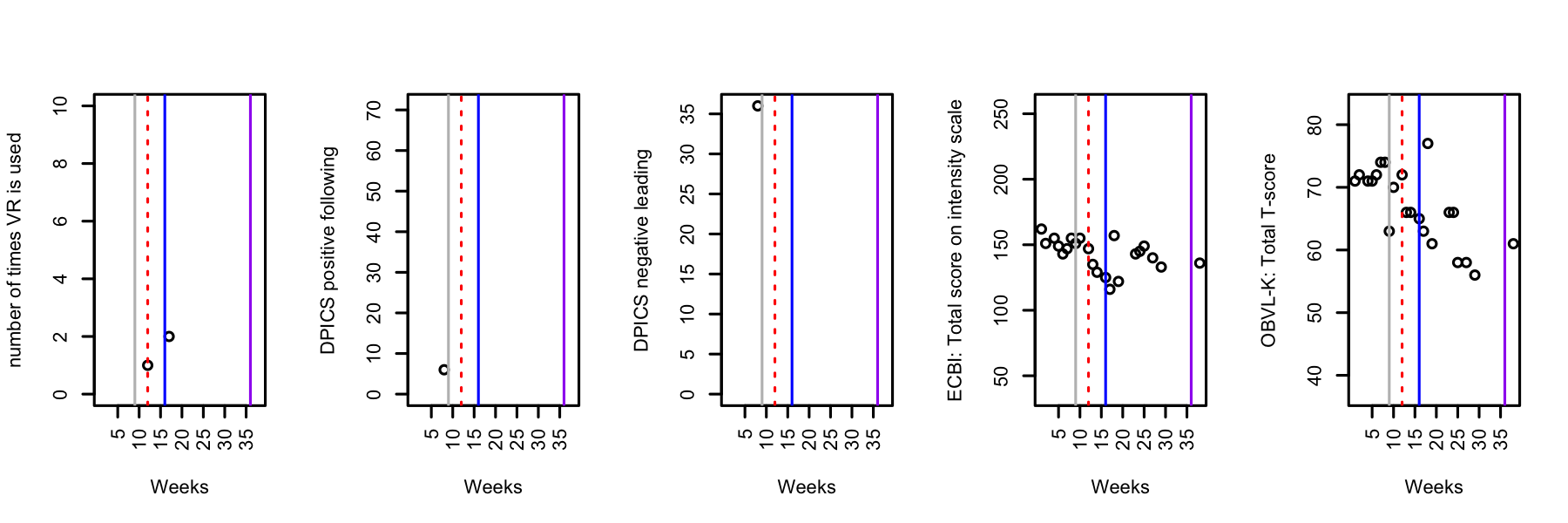
*

*Participant 09*

*
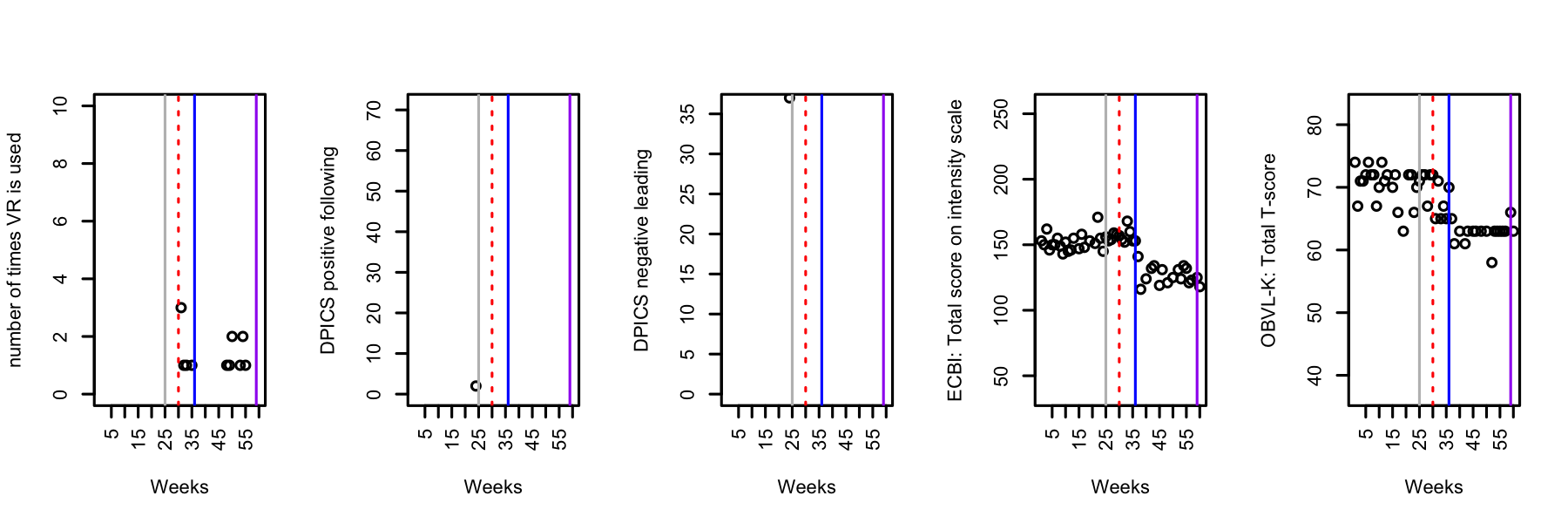

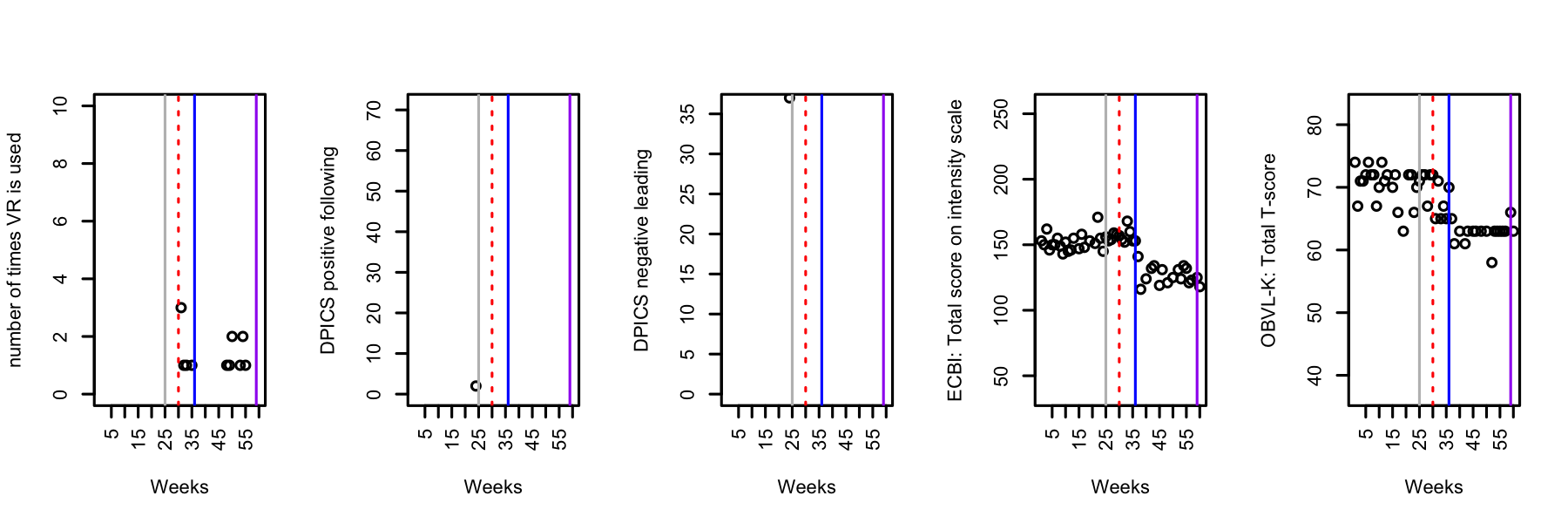
*

*Participant 10*

*
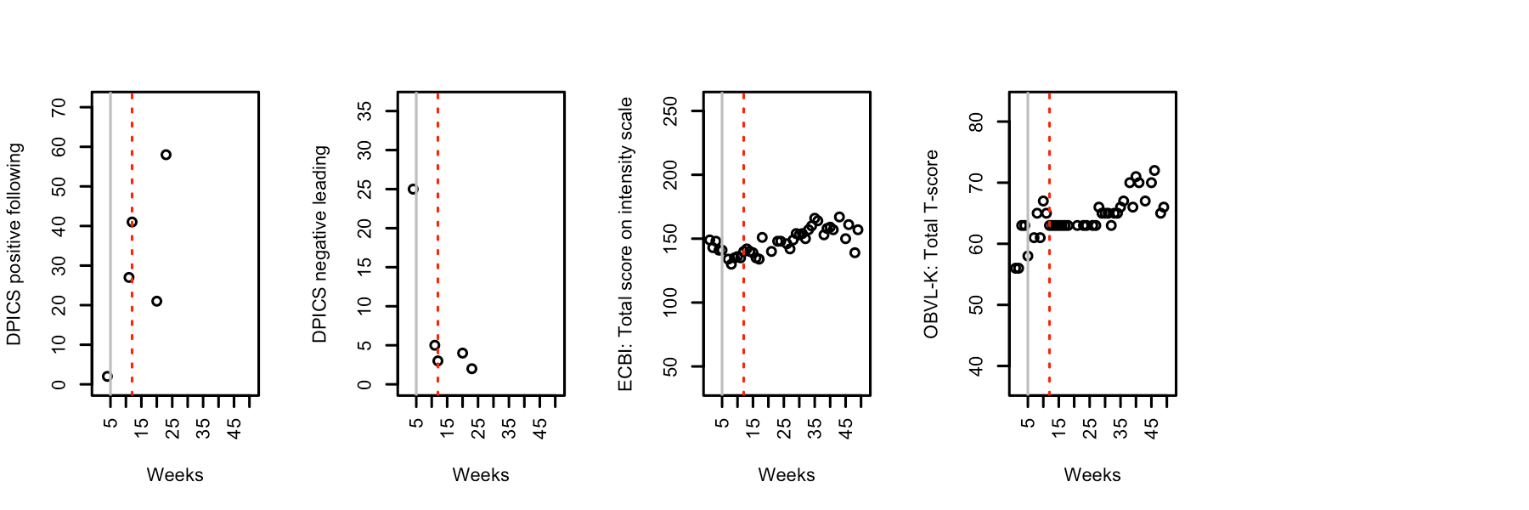
*

*Participant 11*

*
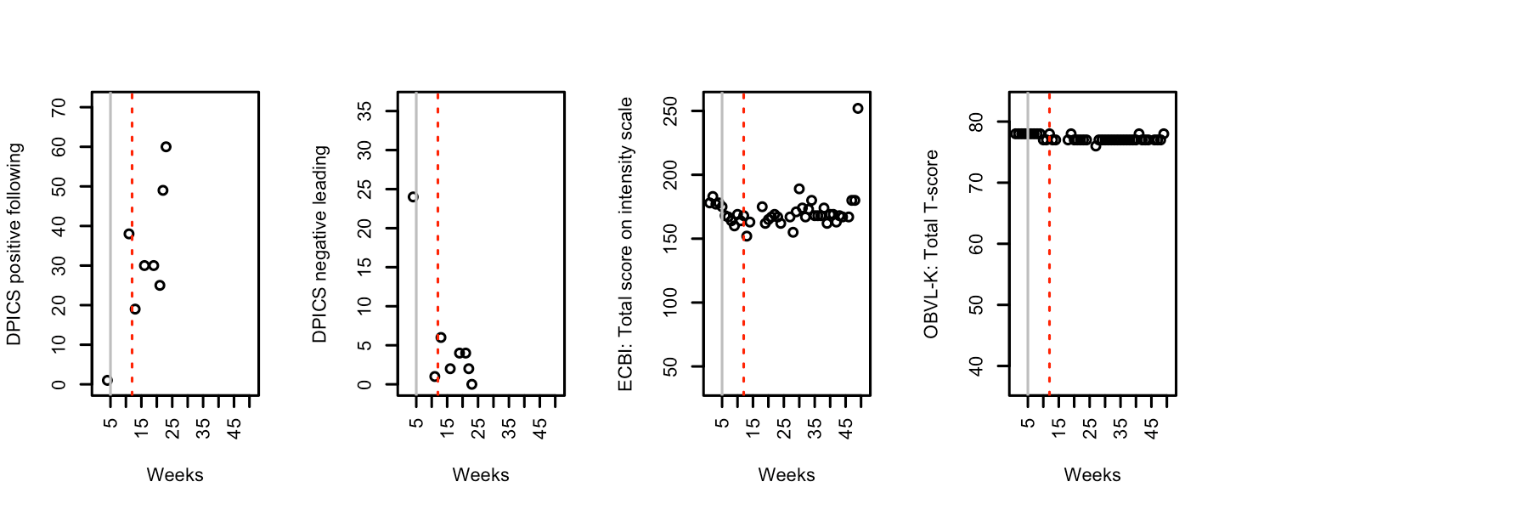
*

*Participant 12*

*
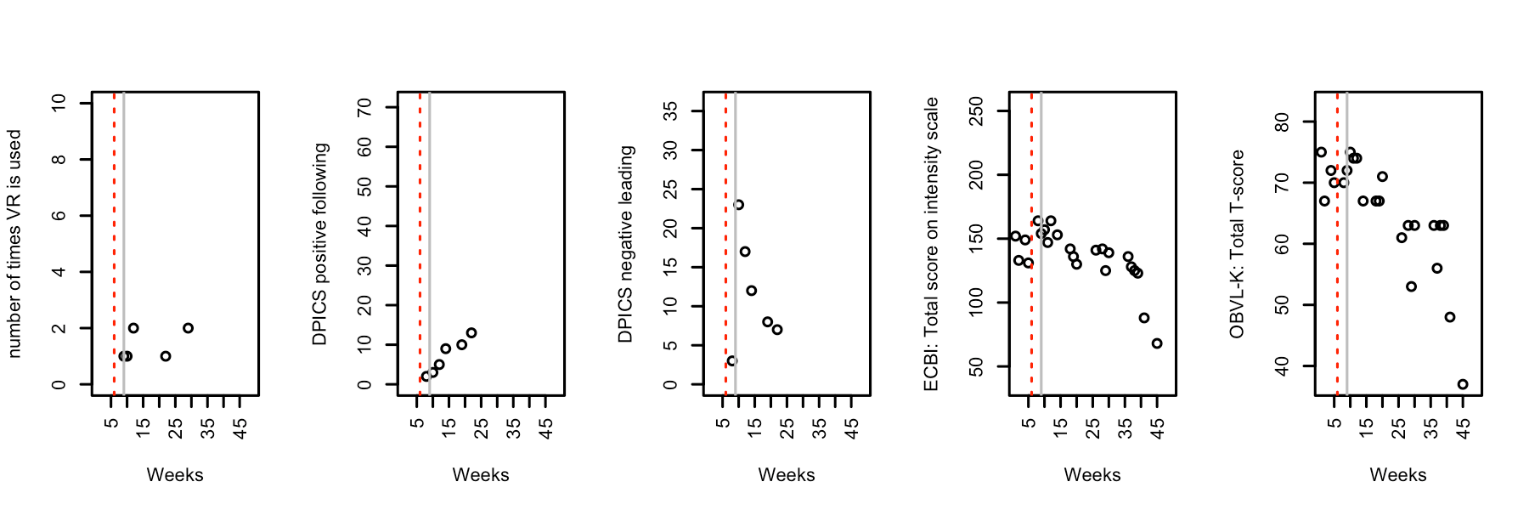
*

*Participant 13*

*
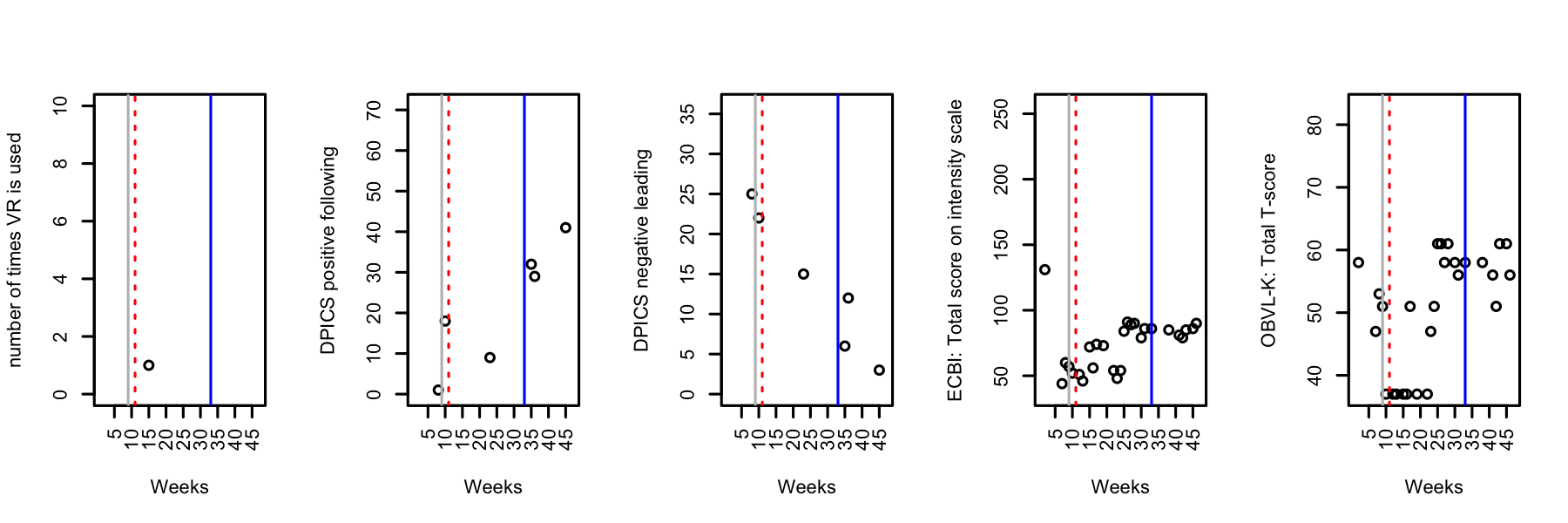
*

*Participant 14*

*
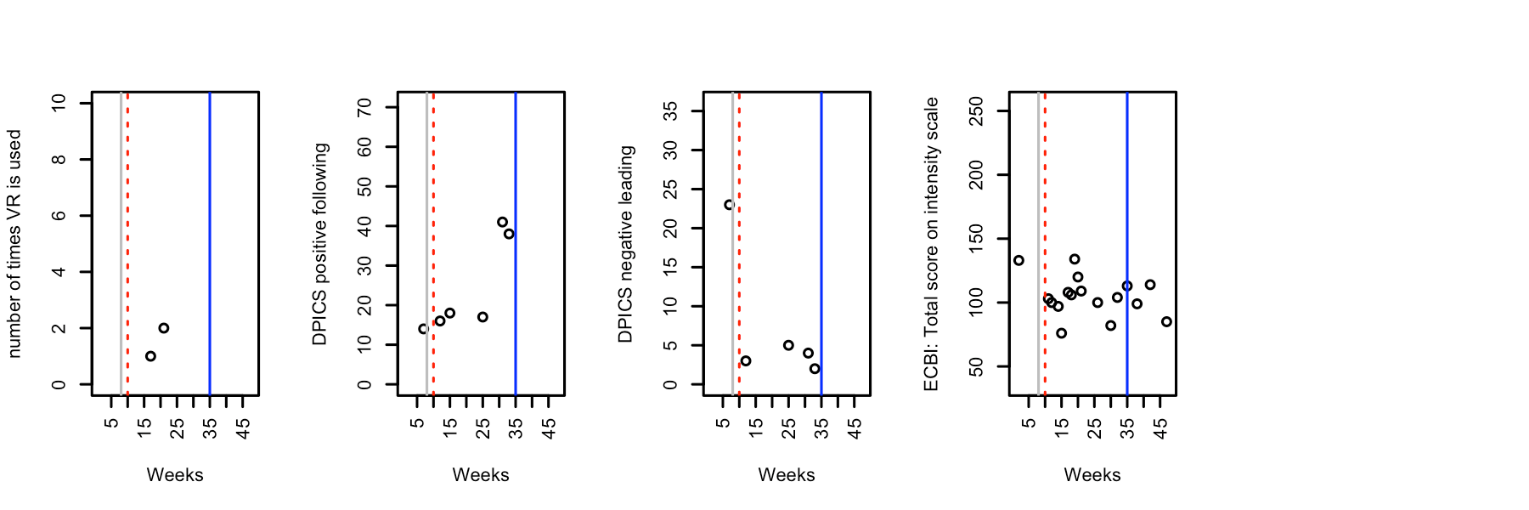
*

*Participant 15
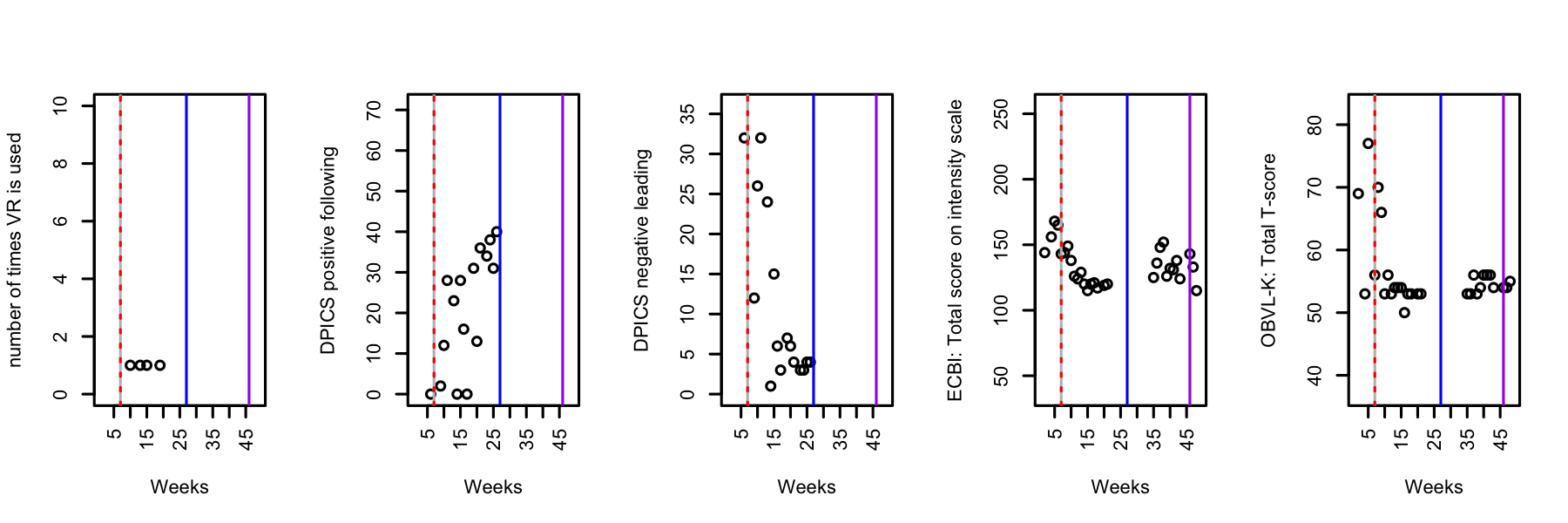
*

*Participant 16*

*
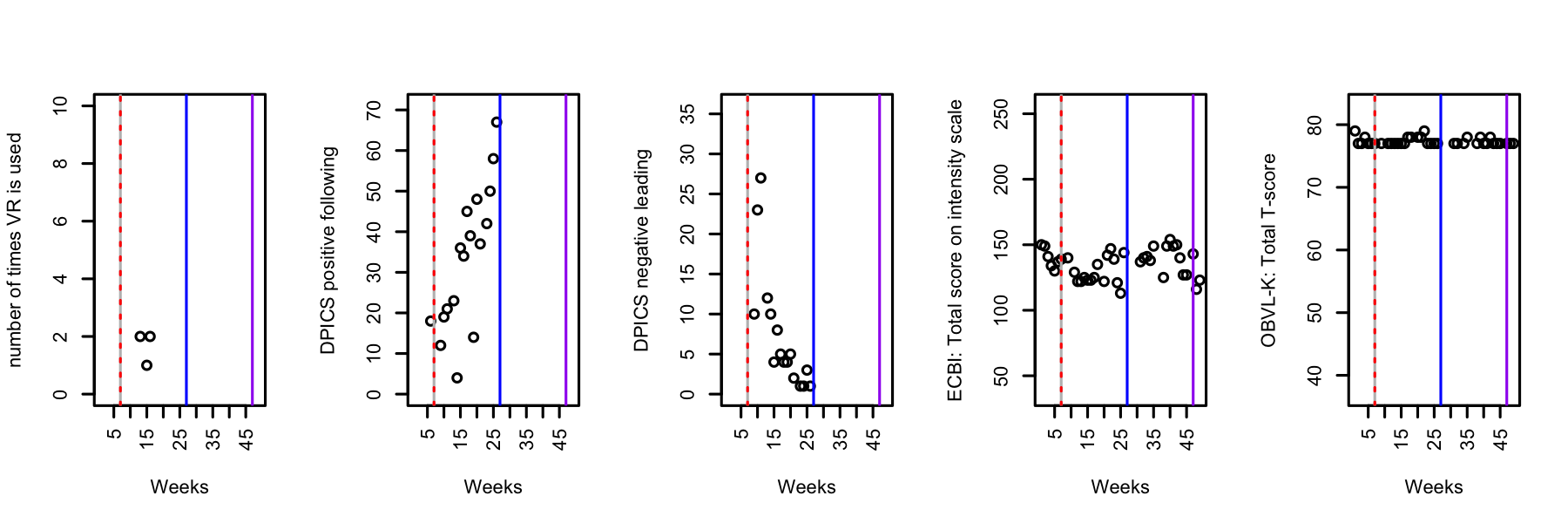
*

*Participant 17*

*
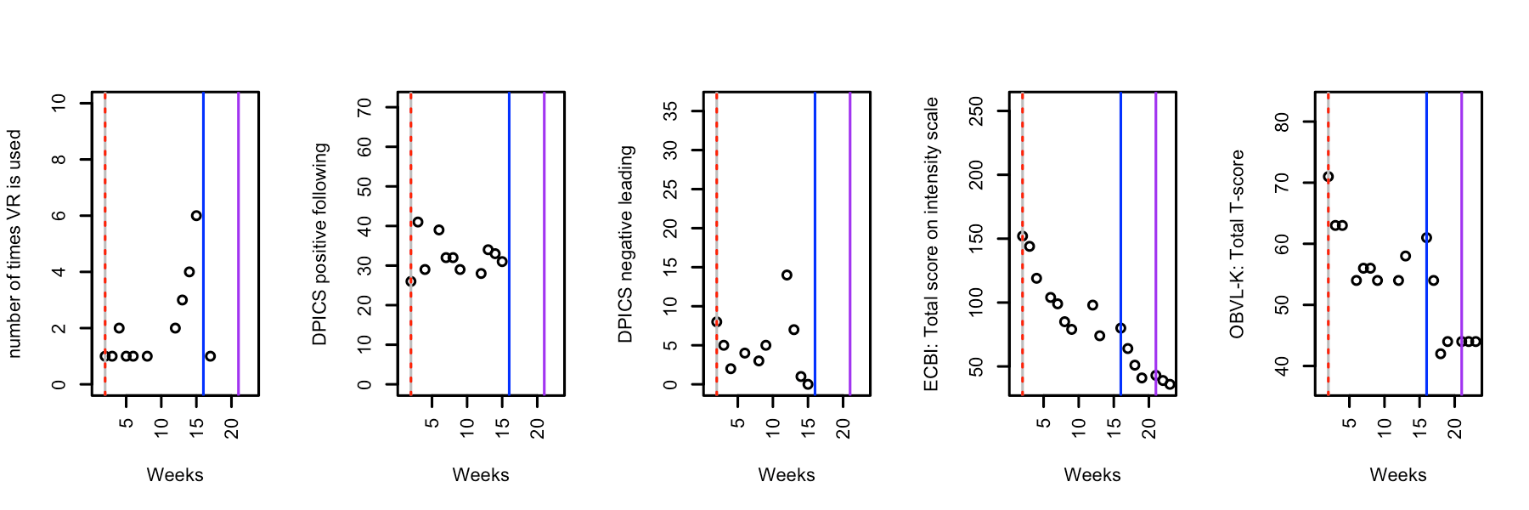
*

*Participant 18*

*
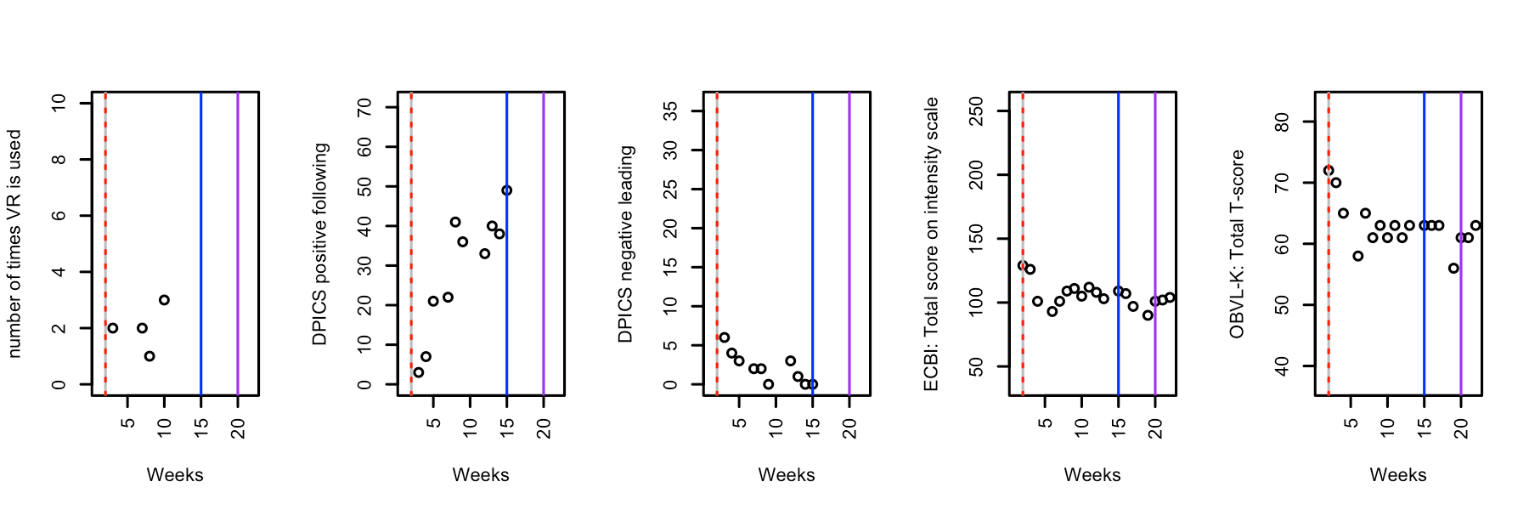
*
