# Supplementary material for: The Added Value of Parents Practicing in Virtual Reality to Illustrate the Use of Innovative Methods in Parent-Child Interaction Therapy: Single-Case Experimental Design
Source: JMIR Pediatr Parent. 2025 Jul 23;8:e60752. doi: 10.2196/60752 (PMC12329383; doi:10.2196/60752)
Supplement: Multimedia Appendix 3 [file pediatrics_v8i1e60752_app3.docx]

Table 6

The total number of times practiced with VR and the raw scores in pre, and posttreatment and follow-up measurements in the DPICS, ECBI and OBVL per participant.

| VR practice group | Participant | VR |  | DPICS | | |  |  | | |  | ECBI | | | | | | |  | OBVL | | |
| --- | --- | --- | --- | --- | --- | --- | --- | --- | --- | --- | --- | --- | --- | --- | --- | --- | --- | --- | --- | --- | --- | --- |
|  |  | Total |  | Positive Following^2^ | | | | Negative Leading^3^ | | |  | Intensity Scale | | |  | Problem Scale | | |  | Total Parenting Stress | | |
|  |  |  |  | T0 | T1 | T2 |  | T0 | T1 | T2 |  | T0 | T1 | T2 |  | T0 | T1 | T2 |  | T0 | T1 | T2 |
| A | 03 | 4 |  | 3 | 89 | - |  | 14 | 0 | - |  | 140 | 143 | 132 |  | 17 | 20 | 21 |  | 72 | 68 | 66 |
|  | 05 | 3 |  | 10 | 23 | - |  | 41 | 57 | - |  | 159 | 161 | 131 |  | 18 | 22 | 5^+^ |  | 79 | 79 | 75 |
|  | 06 | 3 |  | 6 | 28 | - |  | 36 | 19 | - |  | 155 | 109^+^ | 150 |  | 16 | 1^+^ | 14 |  | 75 | 55^+^ | 66 |
|  | 15 | 4 |  | 0 | 11 | 36 |  | 32 | 12 | 8 |  | 165 | 115^+^ | 129^+^ |  | 20 | 0^+^ | 0^+^ |  | 63 | 53^+^ | 48^+^ |
|  | 16 | 5 |  | 18 | 51 | 37 |  | 56 | 10 | 1 |  | 137 | 124^+^ | 135 |  | 15 | 1^+^ | 20 |  | 77 | 76 | 72 |
| B | 02 | 7 |  | 2 | 24 | 51 |  | 5 | 3 | 3 |  | 117^+^ | 63^+^ | 62^+^ |  | 29 | 20 | 0^+^ |  | 56^+^ | 54^+^ | 48^+^ |
|  | 04 | 24 |  | 2 | 17 | 24 |  | 30 | 8 | 10 |  | 123^+^ | 119^+^ | 131 |  | 13 | 17 | 18 |  | 72 | 72 | 76 |
|  | 09 | 14 |  | 2 | 35 | 43 |  | 37 | 22 | 15 |  | 145 | 128^+^ | 128^+^ |  | 21 | 7^+^ | 2^+^ |  | 70 | 68 | 66 |
|  | 12^1^ | 7 |  | 3 | 3 | 1 |  | 51 | 18 | 18 |  | 164 | 88^+^ | 87^+^ |  | 19 | 6^+^ | 7^+^ |  | 72 | 53^+^ | 51^+^ |
|  | 17 | 24 |  | - | 26 | 13 |  | - | 6 | 4 |  | - | 52^+^ | 51^+^ |  | - | 0^+^ | 0^+^ |  | - | 58^+^ | 50^+^ |
|  | 18 | 8 |  | - | 17 | 20 |  | - | 7 | 4 |  | - | 78^+^ | 101^+^ |  | - | 0^+^ | 2^+^ |  | - | 49^+^ | 59^+^ |

Note. VR practice group = VR practice group A practiced with VR less than 6 times; VR practice group B practiced with VR more than 6 times; VR = Virtual Reality; DPICS = Dyadic Parent-Child Coding System; ECBI = Eyberg Child Behavior Inventory; OBVL = *Opvoedingsbelastingvragenlijst*; T0 = pretreatment; T1 = posttreatment; T2 = follow-up. ^1^Dropped out of intervention but remained in study; ^2^Positive Following must increase; ^3^Negative Leading must decrease.
^+^ Raw scores below clinical range are marked with a cross (ECBI Intensity Scale <131 and ECBI Problem Scale <10; OBVL Total T score <60).
